# Applications of Nanotechnology for Spatial Omics: Biological Structures and Functions at Nanoscale Resolution

**DOI:** 10.1021/acsnano.4c11505

**Published:** 2024-12-20

**Authors:** Ruixuan Wang, Waylon J. Hastings, Julian G. Saliba, Duran Bao, Yuanyu Huang, Sudipa Maity, Omar Mustafa Kamal Ahmad, Logan Hu, Shengyu Wang, Jia Fan, Bo Ning

**Affiliations:** † Center for Cellular and Molecular Diagnostics, Tulane University School of Medicine, New Orleans, Louisiana 70112, United States; ‡ Department of Biochemistry and Molecular Biology, Tulane University School of Medicine, New Orleans, Louisiana 70112, United States; § Department of Psychiatry and Behavioral Science, Tulane University School of Medicine, New Orleans, Louisiana 70112, United States; ∥ Groton School, 282 Farmers Row, Groton, Massachusetts 01450, United States; ⊥ St. Margaret’s Episcopal School, 31641 La Novia Avenue, San Juan Capistrano, California92675, United States

**Keywords:** Spatial omics, nanotechnology, microfluidics, nanomaterials, transcriptomics, proteomics, metabolomics, next generation sequencing, mass spectrometry, nucleic acid barcoding

## Abstract

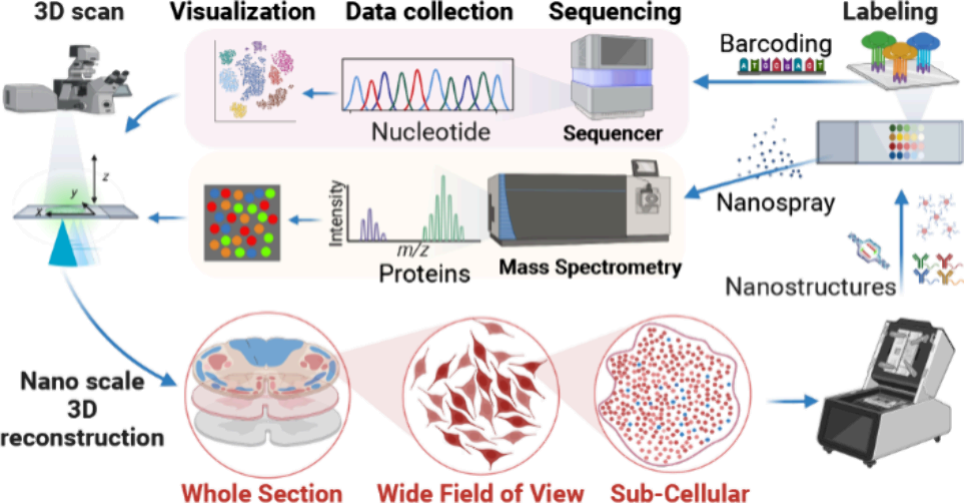

Spatial omics methods are extensions of traditional histological methods that can illuminate important biomedical mechanisms of physiology and disease by examining the distribution of biomolecules, including nucleic acids, proteins, lipids, and metabolites, at microscale resolution within tissues or individual cells. Since, for some applications, the desired resolution for spatial omics approaches the nanometer scale, classical tools have inherent limitations when applied to spatial omics analyses, and they can measure only a limited number of targets. Nanotechnology applications have been instrumental in overcoming these bottlenecks. When nanometer-level resolution is needed for spatial omics, super resolution microscopy or detection imaging techniques, such as mass spectrometer imaging, are required to generate precise spatial images of target expression. DNA nanostructures are widely used in spatial omics for purposes such as nucleic acid detection, signal amplification, and DNA barcoding for target molecule labeling, underscoring advances in spatial omics. Other properties of nanotechnologies include advanced spatial omics methods, such as microfluidic chips and DNA barcodes. In this review, we describe how nanotechnologies have been applied to the development of spatial transcriptomics, proteomics, metabolomics, epigenomics, and multiomics approaches. We focus on how nanotechnology supports improved resolution and throughput of spatial omics, surpassing traditional techniques. We also summarize future challenges and opportunities for the application of nanotechnology to spatial omics methods.

## 1 Introduction

Spatial omics preserves spatial information when evaluating the molecular composition of a specimen, allowing data to be mapped to specific regions, including tissues, single cells, and subcellular regions. Spatial omics helps us better understand cellular organization and interactions in histological landscapes. It can detect molecular parameters in situ, on intact tissue samples having differential transcript or protein abundance within their native spatial context, and it can delineate the interactions between molecular parameters, leveraging different multiplexed labeling methods. Recently reported spatial omics approaches permit in situ spatial profiling of several distinct types of molecular information, including RNA, protein, metabolite, and epigenetic targets. Spatial multiomics methods that simultaneously capture different types of molecular information are now commercially available. Spatial omics applications are increasingly used to answer biological questions in fields ranging from cancer research (especially tumor microenvironment questions) to neuroscience and organismal development.^1−4^

Advances in spatial omics have relied heavily on nanotechnology, since methods that use the intrinsic properties of nanomaterials in nanodevices and nanobiotechnological tools have enabled precise cellular and subcellular labeling and sequencing. As a result, spatial omics methods can now define structure/function down to almost 1 nm versus the 1 μm limit of traditional light microscopy. Methods and techniques adopted from the field of nanotechnology have allowed insight into biological systems at nanoscale resolution, enabling analyses that were not feasible before the use of such nanotechnological approaches.

Spatial omics methods have been reviewed elsewhere,^1−3,5,6^ but few of these articles have focused on the role of nanotechnology in these methods, and described its use in specific applications rather than providing a comprehensive overview of potential applications. For example, one such review described the development and use of nanotechnology tools for the enrichment and omics analysis of circulating cancer cells, highlighting key advances in multiomics liquid biopsy approaches,^7^ while another discussed the use of nanodevice DNA-barcoded fluorescence microscopy for spatial genomics and transcriptomics.^8^

In contrast, this review provides a comprehensive review of the use of nanotechnology in spatial omics, discussing the power and limitations of nanotechnology as applied in recent spatial omics approaches. We first introduce nanotechnology areas relevant to spatial omics applications, including integrated applications of nanomaterials and nanodevices and nanobiotechnology approaches, and highlight key nanotechnologies that have been instrumental in developing spatial omics methods. We then summarize spatial omics methods used to evaluate the global expression of mRNA (spatial transcriptomics), protein (spatial proteomics), metabolites (spatial metabolomics), and epigenetic DNA modifications (spatial epigenomics), and then discuss current multiomics applications (spatial multiomics). Selecting examples from the numerous spatial omics tools, we focus on the advances underscored by nanotechnology (Table 1). We also summarize the critical role of AI and machine learning in spatial omics data processing and the integration of nanotechnology with AI, which has revolutionized spatial biomarker discovery. Finally, we offer a perspective on the remaining challenges and future opportunities regarding nanotechnology for spatial omics applications.

**Table 1:** Current Spatial Omics Methods Applied with Nanotechnology

	Method	Nanotechnology	Advantage	Weakness	Applications	Spatial Resolution	References
Spatial Transcriptomics	EEL FISH	ITO capture surface; DNA barcoding	continuous capture surface; high spatial resolution	lower sensitivity	Sagittal mouse brain sections	200 nm	(63)
	Stereoseq	DNA nanoball-patterned arrays	large field of view; high sensitivity; high spatial resolution	RNA capture limitation; low efficiency	mouse organogenesis spatiotemporal transcriptomic atlas	500 nm	(86)
	scStereo-seq	DNA nanoball-array	single-cell RNA-seq data (∼10-fold greater than Slide-seq)	not applied to tissues	*Arabidopsis* leaves	single-cell resolution (∼30 μm)	(88)
	BARseq	DNA nanoball amplicon; fluorescence labeling; multichannel fluorescence confocal microscopy	multiplexed projection mapping	low spatial resolution; problems for long axons	mouse neuronal projections	cellular resolution (∼150 μm)	(91)
	BaristaSeq	DNA nanoball amplicon; fluorescence labeling; confocal microscopy imaging	amplification efficiency	limited quantity, limited field of view; low spatial resolution, limited to cell culture	BHK cells in culture	cellular resolution (∼150 μm)	(67)
	FISSEQ	DNA nanoball amplicon; fluorescent probes; confocal microscopy imaging	high spatial resolution; 3D visualization	low detection; time-consuming protocol; low sensitivity, limited field of view	human primary fibroblasts	600 nm	(56)
	STAPmap	DNA nanoball amplicon; in situ amplification of a library of cDNA probes	high spatial resolution, 3D visualization	limited in hydrogel-tissue chemistry; limited quantity; limited field of view	mouse cortex from 3D tissue blocks	Subcellular resolution (∼100 nm)	(57)
	Slide-seq	DNA-barcoded beads; confocal microscopy imaging	scalability to large tissue volumes	costly; low transcript detection sensitivity; low spatial resolution	dendritically localized mRNAs of mouse hippocampal neurons	10 μm	(83)
	Slide-seqV2	spatially index barcoded bead arrays	better capture efficiency	low spatial resolution	mouse neocortex	10 μm	(82)
	Seq-Scope	barcode molecule; HDMI-array	speed; straightforward protocol; precise; easy-to-implement; excellent transcriptome capture output; high spatial resolution	limited to capture of the poly-A-tagged transcriptome	portal-central (liver), crypt-surface (colon), and inflammation-fibrosis (injured liver) axes	600 nm	(101)
Spatial Proteomics	LCM	LCM microscopy	high resolution; high yield, and multiplex capability	time-consuming	entomology, agriculture research, embryology, metabolic disease, heart disease, neurobiology, infectious disease, and cancer	Subcellular resolution (∼100 nm)	(107)
	MIBI	metal-labeled antibodies; time-of-flight secondary ion mass spectrometry; Au liquid metal ion gun	high spatial resolution	low protein detection efficiency	tumor microenvironment	200–300 nM	(138)
	CODEX	DNA barcode antibodies; the microfluidics system; fluorescent probe; fluorescence imaging microscope	high spatial resolution; single section and staining; low cost; suitable with common microscope	low protein detection efficiency; requires special reagents and equipment	normal and lupus (MRL/lpr) murine spleens	260 nm	(124)
Spatial Metabolomics	MALDI	crystallized matrix; mass spectrometry imaging	high mass resolution; suitable for examining small samples; reliable results	low spatial resolution; required special preparation steps; vacuum condition	cilia and oral groove in *Paramecium caudatum*; cancer metabolomics; plant metabolites in situ	1.4 μm	(138,74,221,167)
	DESI	nanospray; ionization probe; mass spectrometry imaging	high throughput; ambient operating conditions; no need extra sample preparations; rapid results	low spatial resolution; low sensitivity	cancer metabolomics	10–20 μm	(133,221)
	SIMS	ionization beam; mass spectrometry imaging; metal nanofilm coating	increased spatial resolution; 3D spatial resolution	lower coverage; sensitivity	single-cell tissues	50 nm	(58)
Spatial Epigenomics	Spatial-ATAC-seq	microfluidic deterministic barcoding	ability to capture spatial epigenetic information on tissue	low spatial resolution; data interpretation	mouse embryos	20 μm	(156)
	Spatial-CUT&Tag	in-tissue microfluidic deterministic barcoding	achieve spatial histone modification profiling	low spatial resolution, limited mapping area	the brain of embryonic day 11 (E11) mouse embryos	20 μm	(175)
Spatial Multiomics	NanoString GeoMx DSP	RNA probes, DSP probes; photocleavable DNA tags for oligo detections	high numbers of biomarkers with higher throughput; high RNA detection efficiency	low protein detection efficiency; require manual choice of regions; low spatial resolution	whole tissue sections, FFPE; fresh tissue; fresh-frozen tissue	10–600 μm	(177,178)
	DBiT-seq	PDMS microfluidic chip with DNA barcode; ADTs, optical or fluorescence microscope imaging	in-tissue barcoding approach	low spatial resolution; low protein detection efficiency	mouse embryos	10 μm	(54)
	spatial-CITE-seq	PDMS microfluidic chip with DNA barcode; ADTs; optical or fluorescence microscope imaging	coindexing of transcriptomes and epitopes	low spatial resolution; better protein detection efficiency; poor detection efficiency for low copy number transcripts	whole mouse and human tissue types	cellular resolution (∼150 μm)	(181)
	MOSAICA	DNA probes; fluorescent probe; confocal microscope imaging	3D visualization; high spatial resolution; low cost	limited detection efficiency and accuracy	embryonic, juvenile mouse brain, adult human brain	100 nm	(55)

## 2 Fundamentals of Nanotechnology

Nanotechnology is an interdisciplinary field involving the design, synthesis, characterization, and application of materials, devices, and systems at the nanometer scale (approximately 1 to 100 nm).^3^ This nanometer scale has implications for various fields including materials science, electronics, energy, environmental science, engineering, and medicine.^9^ Its broad-spanning implications are attributed to the physical, chemical, and biological properties that emerge following control of nanostructure parameters such as shape and size.^10,11^ These properties, distinct from properties of the same materials at the microscopic or macroscopic scale, are not evident without nanotechnology’s larger surface area-to-volume ratios and quantum effects.^9,12−14^ Nanotechnology includes synthetic and natural nanostructures and encompasses both bottom-up assembly and top-down fabrication techniques.^15−18^ For example, three different elements combine to make indium tin oxide, and though its bulk form is yellowish/gray in color and scatters light, when layered in <100 nm sheets, it is optically transparent. This optical transparency coupled with its electrical conductivity lends indium tin oxide to practical applications, such as LED displays.

Nanotechnology has broad applications for spatial omics methods and biomedicine.^19^ Nanomaterials exist within the same size domain as subcellular organelles and biological macromolecules, giving them the ability to exhibit similar functionality at the biomolecular level. These include nano-objects, such as polymeric nanoparticles, gold nanorods, and quantum dots. Specifically, gold nanorods are used to enhance signals in spatial transcriptomics to amplify signals from low-abundance biomolecules.^20,21^ Moreover, nanostructured materials, such as carbon nanotubes, nanodiscs, and nanocrystals, are also used to enhance spatial resolution in both spatial omics and biomedicine.^22−27^ These nano-objects and nanostructured materials have aided the functionality of microfluidic devices to improve sample throughput and enhance characterization of biomolecular structures at the nanometer level within instruments such as nucleic acid sequencers, mass spectrometers, and confocal microscopes.^28^ Nanobiotechnology refers to structures derived from biological macromolecules, such as DNA, proteins, or lipids, and may be self-assembled; examples are liposomes, fluorescently labeled DNA, and antibody technologies.^18,21,29,30^

### 2.1 Integrated Applications of Nanomaterials and Nanodevices

Nanomaterials, products of chemistry and classical materials engineering, are defined by their size: either one dimension of the material, or a single unit within, measures between 1 and 100 nm. Under this broad definition, nanomaterials can be distinguished by their elemental composition. Careful manipulation of the elemental composition allows the nanomaterial to be tailored to the application of interest. But nanomaterials can also be distinguished by their dimensionality, being categorized as zero-, one-, two-, or three-dimensional,^31,32^ and nanomaterials composed of even a single element can produce different properties as a function of their dimensionality. For example, zero-dimensional fullerenes (i.e., “bucky balls”) have different properties than one-dimensional nanotubes, which are distinct from two-dimensional (2D) graphene sheets and three-dimensional (3D) diamond, despite all being composed of pure carbon. In addition to fullerenes, zero-dimensional nanomaterials include spherical nanoparticles and quantum dots. Higher-dimensional nanomaterials have been designed for advantageous mechanical or chemical properties. One-dimensional nanomaterials provide classic examples of this, carbon nanotubes feature enhanced tensile strength and gold nanorods boast increased electrical conductivity. Nanomaterials that are 2D and 3D are even more complex, generated to leverage the properties that emerge from increased surface area-to-volume ratios, such as the optical properties of indium tin oxide nanolayers.

Nanodevices, also referred to as nanotools, are divided into two broad classes. The first broad class allows scientists to characterize inorganic and organic systems with nanometer resolution. The confocal microscope is a prototypical nanodevice in this class. Confocal imaging stems from refining the source light to a pinpoint and visualizing an object after the light transits through a pinhole. When combined with fluorescence, this optical advance has increased the resolution of imaging below the 1 μm threshold of the classical light microscope. Although initially applied to materials science, the combination of higher resolution and diminished photobleaching effects makes confocal fluorescence microscopy ideal for biological applications, including the imaging of tissues and live cells. Other nanodevices in this class assist in analyzing complex materials and mixtures to identify individual nanoscale components. Most popular among these tools is mass spectrometry, which involves desorbing molecules from a sample via ionization and determining their identity by examining mass-to-charge ratios. High sensitivity variations of mass spectrometry exist, such as Secondary Ion Mass Spectrometry (SIMS), which can identify components of organic and inorganic materials and mixtures with resolution as low as 50 nm.^33,34^ Quantitative mass spectrometry has also been used to identify protein–protein interactions within cell extracts, especially after enrichment by antibody-mediated affinity purification.^35,36^ The second broad class of nanodevices comprises nanoscale systems that increase the efficiency of biological or chemical reactions. One such nanodevice is the microfluidic chip, which has miniaturized chambers or channels and can be used to carry out biochemical reactions or separations as a result of fluidic properties specific to nanoscale dimensions. Such nanodevices have been used for the detection and analysis of various biochemical and biological targets, including DNA, proteins, molecules, and viruses.^37−43^

### 2.2 Nanobiotechnology

Nanobiotechnology describes the interface between nanotechnology and biology. One established example of nanobiotechnology is the liposome, a nanometer-sized lipid structure composed of a lipid bilayer surrounding an aqueous environment. Within this aqueous environment, groups of biologically relevant molecules can be sequestered, stored, and delivered. Liposomes have been instrumental for carrying nanosized cargo—they were the engineered nanoparticles used for drug delivery^15^—and for enclosing nanosized chemical reactions, as has been done in microfluidic chips.

A second established example of nanobiotechnology is DNA nanotechnology, which uses engineered duplex DNA strands as the nanoscale engineering material.^16,44−47^ One type, structural DNA nanotechnology, uses DNA as a physical material unit for the self-assembly of nanoscale structures.^18,29^ Another type, dynamic DNA nanotechnology, is focused on reconfigurable and autonomous devices such as the amplification approach hybridization chain reaction, which uses secondary loop structure hairpin DNA monomers as an energy source.^46,48−50^ In this type, DNA probes labeled with a fluorescent molecule are used for pathogen detection, protein detection, and nanoscale imaging.^21,30^ A third established example is a device or tool that incorporates nanosized, membrane-spanning protein channels, which exhibit the nanofluidic phenomena.^28^ These protein channels can be designed to allow the transit of specific molecules through the narrow, nanometer-wide pore due to osmotic drivers, and coupling the transit of specific biomolecules to changes in ion movements (ie, currents) allows for the detection of specific molecular transit events. This can be used to distinguish nucleotides, as in nanopore sequencing.

## 3 Key Nanotechnologies for Spatial Omics

**Figure 1:**
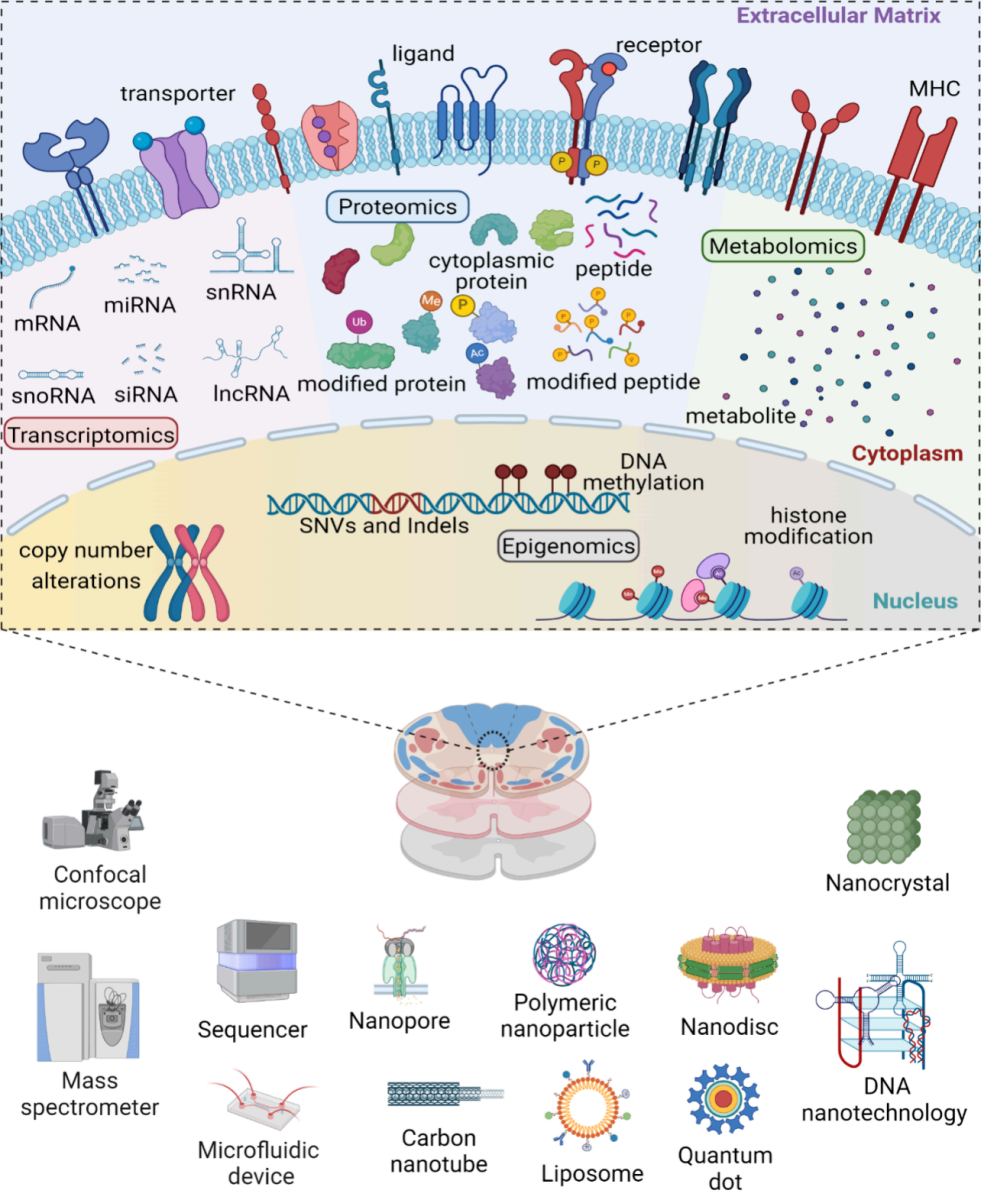
Spatial omics provide information about transcriptomics, proteomics, metabolomics, and epigenomics while preserving spatial information, such as subcellular localization. Examples of nanotechnology for biological applications are shown. Abbreviations: mRNA: mRNA; miRNA: microRNA; snRNA: small nuclear RNA; snoRNA: small nucleolar RNA; siRNA: small interfering RNA; lncRNA: long noncoding RNA; SNV: Single-Nucleotide Polymorphism; MHC: Major Histocompatibility Complex. The figure was created with BioRender.

Nanotechnologies, described generally in the previous section, have supported the construction of spatial omics methods.^51,52^ In the following section, we define spatial omics and describe several specific nanotechnologies that have been crucial for the development of spatial omics methods. Spatial omics provides global biomolecule information—including data from transcriptomics, proteomics, metabolomics, and epigenomics—layered onto a histological landscape. In other words, spatial omics provides omics information while preserving spatial information at sufficient resolution for each application (Figure 1).^1,3,53,54^ The resolution of spatial omics has improved over time, from the 1 μm limit of traditional light microscopy to almost 1 nm. Spatial multiomics are also possible, with current technologies allowing for the simultaneous evaluation of two or more biomolecular domains.^55^ An example is the simultaneous evaluation of transcriptomics and proteomics via subcellular views of global RNA and protein overlaid onto 3D histological structures.^56−58^

### 3.1 Nanodevices for Spatial Omics

Nanodevices support spatial omics by providing nanoscale compartments that can recapitulate physiologically relevant cellular activity while also increasing throughput with parallel reactions. Microfluidic devices are one example, employed in the spatial multiomics methods Deterministic Barcoding in Tissue for spatial omics sequencing (DBiT-seq) and Spatial Assay for Transposase-Accessible Chromatin and RNA using Sequencing (spatial-ATAC-seq). In DBiT-seq, channels in a microfluidic chip demarcate regions on a tissue section, ultimately creating a series of separate assays to define tissue pixels for protein or RNA detection. Other examples are microfluidic valve-, droplet- or nanowell-based technologies, which are emerging in the field of single-cell transcriptomics due to their superior ability to capture and process single cells and their components.^59,60^ Nanoscale droplets can also contain specific biological components to perform numerous parallel biochemical reactions within a small volume. For example, Macosko et al. developed a microfluidic single-cell transcriptomics platform called Drop-seq, which brings single cells and barcoded beads together in nanoliter droplets, allowing numerous biological assay outputs from a single small-volume reaction.^61,62^

### 3.2 Advanced Imaging Techniques in Spatial Omics

Confocal microscopy provides high-resolution image localization suitable for nanoscale imaging in spatial omics. The principle of confocal imaging involves refining source light to a pinpoint and visualizing resultant fluorescent images after the light passes through a pinhole, thereby increasing the resolution beyond the 1 μm threshold of classical light microscopy. This enhanced resolution is ideal for imaging tissue and is widely employed in spatial omics methods such as Enhanced ELectric Fluorescence in situ Hybridization (EEL FISH), Multi-Omics Single-scan Assay with Integrated Combinatorial Analysis (MOSAICA), and Spatially Resolved Transcript Amplicon Readout Mapping (STARmap).

### 3.3 Mass Spectrometry in Spatial Omics

MS is a fundamental analytic method used in spatial omics, particularly spatial proteomics, for high-resolution protein localization. MS characterizes a sample by ionizing, separating, and detecting its components before quantifying the abundance of the charge/mass values (*m*/*z*). Quantitative MS has been employed to identify protein–protein interactions within cell extracts, especially following enrichment by Antibody-Mediated Affinity Purification–MS (AP–MS) experiments.^35,36^

### 3.4 DNA Nanotechnology in Spatial Omics

DNA molecules form the basis of numerous nucleic acid detection strategies within spatial omics. For example, fluorescently labeled DNA probes enable single-molecule nucleic acid detection when coupled with super-resolution microscopy, forming the foundation for methods like EEL Fish, Fluorescent in situ RNA Sequencing (FISSEQ), and MOSAICA.^55,56,63^

Employing DNA as a barcode is critical for increasing multiplex detection capability and essential for investigating complex biomolecular domains in omics research. DNA barcodes comprise a specific DNA sequence that does not naturally occur in the examined species. By attaching these barcodes to DNA probes, these DNA barcodes can be typed and coupled to next generation sequencing technology for detection. More specifically, they facilitate detection of binding events to complementary target sequences, enabling multiplexed detection by creating numerous independent labels in parallel.

DNA barcodes are also the mainstay of regional biomolecule labeling, allowing precise identification and localization of molecules within a sample. DNA barcodes added to single molecules by ligation or polymerase-catalyzed events direct the creation of a specific nm-spaced grid. DNA location detection barcodes, each having precise location sequence addresses, are added to distinct confined locales in series, allowing for the identification of the original DNA. Adding the DNA location detection barcodes can be accomplished with the help of microfluidics or other technologies. DNA barcodes for multiplex detection and spatial mapping have a central role in Barcode in situ Targeted Sequencing (BaristaSeq), Seq-Scope, DBiT-seq, Spatial Co-indexing of Transcriptomes and Epitopes for Multi-Omics Mapping by Highly Parallel Sequencing (spatial-CITE-seq), and Spatial Cleavage Under Targets and Tagmentation (spatial-CUT&Tag).

Detecting individual nucleic acid locations is a facet of spatial omics, and increasing the sensitivity of target detection can be accomplished with nanometer-sized DNA balls (DNA nanoballs). DNA nanoballs comprise thousands of copies of a specific sequence, which are produced by Rolling-Circle Amplification (RCA). RCA is an isothermal nucleic acid amplification method widely applied for the in vivo imaging of various targets, including messenger RNA (mRNA), double-stranded DNA (dsDNA), microRNA (miRNA), and proteins.^64,65^ RCA utilizes a circular DNA template and special DNA or RNA polymerases to produce a rolony (ie, a rolling circle colony) containing thousands of copies of the original sequence, termed a DNA nanoball, < 1 μm in size.^17,64,66^ The sequences of rolonies in the amplifying and sequencing mRNAs for in situ approaches are read out by sequencing by ligation.^67^

Expanding the breadth of nucleic acid detection capability in spatial omics, many bioanalytical applications use RCA-based platforms that combine RCA with DNA-zymes, aptamers, and nanozymes to form the basis for in situ sequencing technologies.^68−71^ RCA can locally amplify specific nucleic acid sequences, and its ability to detect single molecules directly in cells and tissues makes it ideal for in situ imaging, revealing critical biological processes. For instance, it has been widely used for imaging the spatial location of specific mRNAs within single cells.^72^ Related approaches using DNA or RNA barcodes achieve cellular resolution for cell lineage tracing, as in neuronal projection mapping. In situ sequencing approaches that combine RCA with cellular address barcodes achieve high throughput without sacrificing spatial resolution. In situ sequencing is the basis for BaristaSeq, STARmap and Spatial Enhanced Resolution Omics-Sequencing (Stereo-Seq).

## 4 Nanotechnological Applications in Spatial Omics Approaches

After discussing nanotechnology in general and providing specific examples of key nanotechnologies for spatial omics, we now turn our discussion to spatial omics methods, many of which rely on the key nanotechnologies we have already mentioned. In the following sections, we discuss spatial omics methods that evaluate mRNAs (spatial transcriptomics), proteins (spatial proteomics), biological metabolites (spatial metabolomics), and epigenetic marks (spatial epigenomics) and subsequently mention multiomics applications (spatial multiomics), providing examples within each biomolecular domain. For each method, we describe how it works, give some examples of how it has been used, discuss its advantages and limitations, and mention its underlying nanotechnology.

### 4.1 Spatial Transcriptomics

Highlighted as Method of the Year in 2020 by Nature Methods,^73^ spatially resolved transcriptomics combines transcriptome-wide RNA sequencing with histology-based images to precisely map RNA expression and thereby provide further insights into the cellular transcription of biological systems.^1,3,74^ Spatially resolved transcriptomics can elucidate single-cell nucleic acid expression throughout entire solid tissues or organs while preserving spatial subcellular localization.^5,75,76^

Spatial transcriptomics was accomplished by Laser Capture Microdissection (LCM).^3,5^ In LCM, a laser precisely dissects a microscopic region (eg, a single cell), which may be input into high-throughput RNA sequencing (LCM-seq). This method produced gene expression profiles within defined ∼10 μm compartments on Formalin-Fixed Paraffin-Embedded (FFPE) tissue sections, distinguishing RNAs in tumor cells from normal adjacent cells to reveal important molecular events in cellular oncology.^77^ Later, image-based spatial transcriptomics was accomplished by Single-Molecule RNA Fluorescence in situ Hybridization (smFISH). This technique uses super-resolution microscopy to facilitate the acquisition of high-resolution images (10 to 20 nm).^78,79^ EEL FISH, a derivative of smFISH, combines multiplexed RNA detection with high-resolution, large-area imaging and generates faithful RNA quantitative maps that retain spatial cellular information.^63^ Spatial barcoding-based transcriptomics like BaristaSeq, STARmap, and FISSEQ layer engineered nucleic acid tags onto in situ sequencing technologies and use DNA nanoballs to amplify the specific detection signals for imaging.^56,57,78^ The following sections review several tools available for spatial transcriptomics.

#### 4.1.1 Enhanced Electric Fluorescence In Situ Hybridization

**Figure 2:**
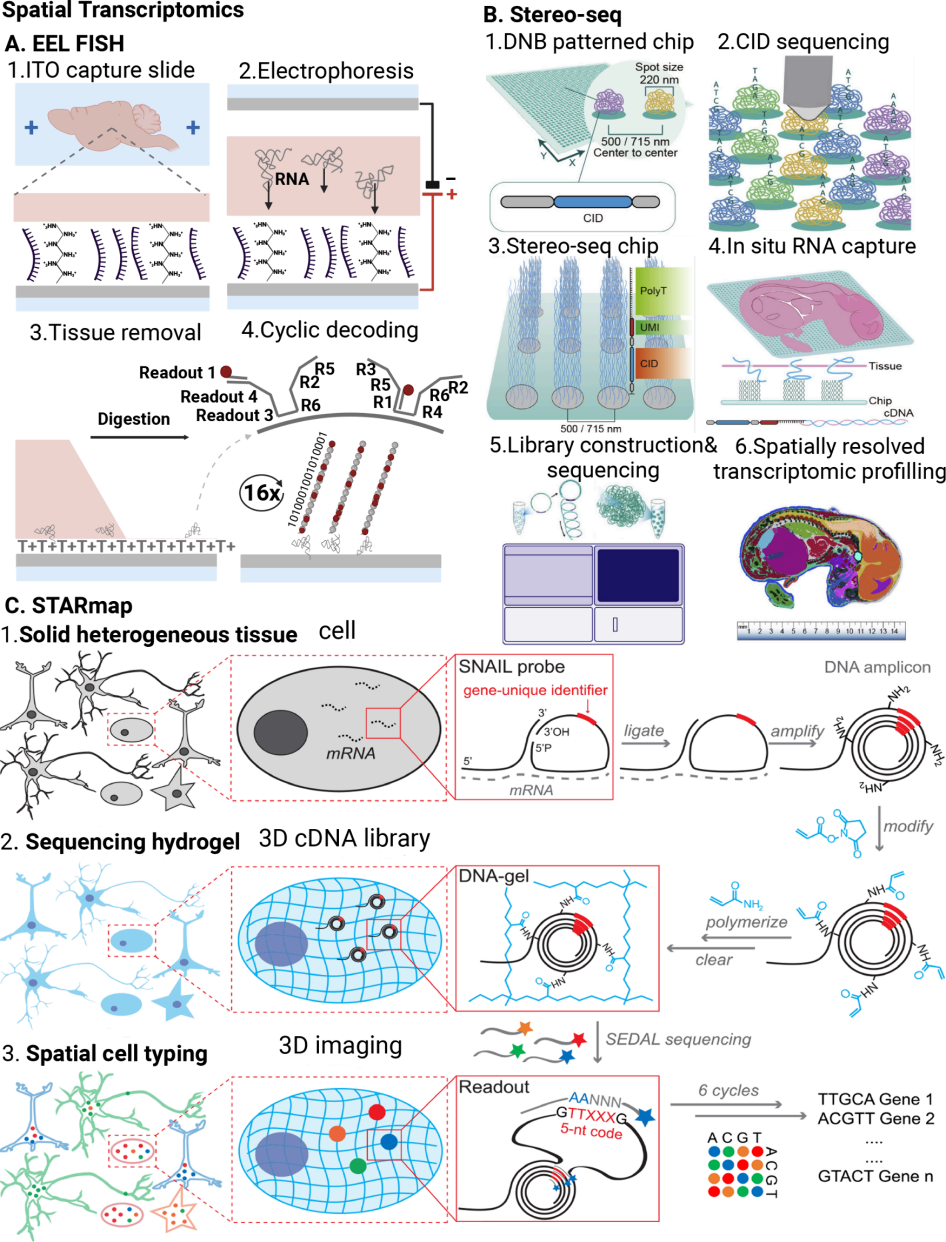
Highlighted spatial transcriptomics methods. A. Schematic illustration of the EEL FISH protocol,^63^ which involves RNA transfer by electrophoresis, capture on an ITO slide, tissue removal, and cyclic fluorescent probing and subsequent decoding. B. The Stereoseq workflow.^86^ First, the DNB-patterned array chip is designed. Then, in situ sequencing determines spatial coordinates of specifically barcoded oligonucleotides. Next, capture probes are prepared by ligating UMI-polyT containing oligonucleotides to each DNB spot, followed by in situ RNA capture from tissue and cDNA amplification, library construction, and sequencing. Finally, the data is analyzed. C. STARmap workflow.^57^ The method integrates hydrogel-tissue chemistry and targeted signal amplification, with 3D in situ transcriptomics using intact tissue. Part A was adapted with permission under a Creative Commons CC-BY license from ref (63). Copyright 2022, published by Springer Nature. The figure was created with BioRender. Part B was reproduced with permission under a Creative Commons CC-BY license from ref (86). Copyright 2022 published by Elsevier Inc.. Part C was reproduced with permission from ref (57). Copyright 2018 The American Association for the Advancement of Science. Abbreviations: EEL FISH: Enhanced Electric Fluorescence in situ Hybridization; ITO: Indium Tin Oxide; Stereoseq: Spatial Enhanced Resolution Omics-Sequencing; DNB: DNA nanoball; CID: Coordinate Identity; UMI: Unique Molecular Identifiers; STARmap: Spatially Resolved Transcript Amplicon Readout Mapping; 3D: three-dimensional.

EEL FISH is a spatial transcriptome profiling method that employs a set of combinatorial, binary barcode tags to detect RNA overlaid onto a histological image.^63^ EEL FISH electrophoretically transfers RNA from a tissue section onto a nanosurface coated with an optically transparent and electrically conductive layer of indium tin oxide (Figure 2A).^63^ Electrophoretically transferring RNA is superior to transferring RNA by passive diffusion, as is done in sequencing-based methods, due to the preservation of RNA localization.^80−83^ In EEL, after residual tissue removal, the result of the transfer is a collapsed 2D grid of mRNA on the coated nanosurface with precise in situ spatial information (Figure 2A). Next, a set of probes, tagged by combinations of 40 labeling barcodes, are used for 16 rounds of imaging (Figure 2A).^63^ After fluorescent decoding and encoding of binary label addresses, barcode identities define locations for numerous mRNAs within a single fluorescence capture field,^63^ which can be matched to an image of the original histological section. One example of EEL FISH is its application to sequential sagittal sections of mouse brain to measure the expression of 440 genes, highlighting complex RNA expression patterns that lie underneath tissue organization.^63^ Despite its advantages, EEL has lower sensitivity and resolution than other tissue-based smFISH methods.^84,85^ (The resolution of EEL FISH, defined by the diffraction-limited imaging resolution of the fluorescent label, approaches 200 to 400 nm.) Future improvements, such as maintaining RNA stability, magnifying the capture field, and expanding barcode detection, could refine EEL sensitivity and resolution to match smFISH for single-molecule imaging.^84,85^ Nanotechnologies that support EEL include the indium tin oxide capture surface and use of combinatorial DNA barcoding to tag numerous mRNAs—the latter a rudimentary example of nanocomputing.

#### 4.1.2 Spatial Enhanced Resolution Omics-Sequencing

Stereoseq is another strategy for spatial transcriptomics (Figure 2B). Stereoseq creates a grid of spots on a lithographically etched nanofluidic chip, the grid acting as a capture surface for mRNAs from a tissue section.^86^ Each DNA nanoball spot is 220 nm in diameter and the spacing between centers of two adjacent nanoballs is 500 or 715 nm (Figure 2B). The DNA nanoball–patterned array chip has 400 spots per 100 μm^2^ to define the pixel size. After a tissue section is laid onto the chip, the DNA nanoballs capture mRNAs and, following a second round of rolling circle amplification, create a library of mRNAs from the original source, which have been sorted to contain specific regional labels defined by the DNA nanoball grid (Figure 2B).^86^ Stereoseq was used to create the Mouse Organogenesis Spatiotemporal Transcriptomic Atlas (MOSTA), which defines detailed topographical information about the stepwise emergence of tissue-specific cell identities during organogenesis.^86^ Stereoseq has been performed to capture spatially resolved single-cell transcriptomes of axolotl telencephalon sections during development and regeneration.^87^ Despite the method having genome-wide coverage, Stereoseq has limited sensitivity and may fail to detect the low copy numbers of RNAs from low-expression genes. Stereoseq also has trouble distinguishing single cells from a mixture of multiple similar cell types, especially smaller cell types like immune cells. A updated version, Single-Cell Stereo-Seq (scStereo-seq), utilizes spatial transcriptomics and plant cell wall staining onto histological cell–cell boundaries, allowing in situ single-cell transcriptome profiling in mature *Arabidopsis* leaves.^88^ The nanotechnology elements of Stereoseq are DNA nanoballs coupled to precise spacing on a nanofluidic capture surface and next-generation sequencing.

#### 4.1.3 Barcode Anatomy Resolved by Sequencing

Barcode Anatomy Resolved by Sequencing (BARseq) is a multiplexed and high-throughput method for mapping neuronal projections at cellular resolution. BARseq combines Multiplexed Analysis of Projections by Sequencing (MAPseq) and in situ sequencing of cellular tagging barcodes.^56,67,89,90^ MAPseq is a technique for mapping neurons by labeling large sets of neurons with barcodes (random RNA sequences).^89^ The advantage of BARseq is its ability to match nearby cortical areas with distant subcortical projections by relying on specific barcode sequences that functionally transit through neuronal projections.^91^ Unlike conventional optical approaches to mapping projections, BARseq relies on matching barcodes without errors over distance, and is, therefore, superior to other multiplexed optical tracing methods.^92^ Theoretically, BARseq can label tens of millions of neurons in a single experiment without a specialized high-speed microscope because of the combinatorial diversity provided in the barcode design; for example, a 30-nucleotide (nt) sequence set can generate about 4^30^ to 10^18^ barcodes. Moreover, the spatial resolution of BARseq approaches subcellular dimensions, sufficient to resolve the organization of projections across neuronal subtypes. The spatial resolution of BARseq may be even further improved with LCM or direct in situ sequencing of projection barcodes.^93^ BARseq mapped the projections of 3,579 neurons to 11 areas in the mouse auditory cortex and confirmed the laminar organization of the three top classes of projection neurons (intratelencephalic, pyramidal tract-like, and corticothalamic). Nanotechnologies that make BARseq possible include DNA nanoballs amplified by RCA, fluorescent labels of nucleotides, and multichannel fluorescence confocal microscopy.

#### 4.1.4 Barcode In Situ Targeted Sequencing

BaristaSeq was published in 2017.^67^ It is a modified version of the gap padlock probe–based method for in situ barcode sequencing compatible with Illumina sequencing chemistry and is suitable for barcode-assisted lineage tracing and mapping for long-range neuronal projections.^67,91^ BaristaSeq uses reverse transcription to convert an RNA barcode sequence into complementary DNA (cDNA) followed by hybridization of a padlock probe and gap-filling ligation to create circular RCA templates.^91,94^ Two distinct fluorescent probes, each recognizing a bracketing padlock, are used during the gap-filling ligation steps. Fluorescence is evaluated to detect probe pairs targeting the diluted padlock after rolony generation, and Illumina chemistry is applied to sequence the samples and determine probe identities. A spinning disk microscope and laser scanning confocal microscope are then employed to image the sequencing. The accuracy and efficiency of BaristaSeq was demonstrated by sequencing random barcodes (15-nt barcode set) expressed in cultured Baby Hamster Kidney (BHK) cells.^89^ BaristaSeq increased the amplification efficiency by 5-fold, and this was coupled with high sequencing accuracy (>97%) compared with other in situ sequencing techniques. BaristaSeq also has limitations; it has only been applied to cultured cells, and its resolution is limited to the cellular, not subcellular level. Nanotechnologies applied in BaristaSeq are DNA nanoballs that function to amplify detection signals, fluorescent probes used for the gap padlock detection step, and the confocal microscope for imaging.

#### 4.1.5 Fluorescent In Situ RNA Sequencing

FISSEQ was proposed in 2003 to selectively amplify DNA on a solid substrate, allowing for targeted genome and transcriptome sequencing.^95−98^ The next generation of FISSEQ provides transcriptome-wide in situ RNA evaluation across multiple specimen types and spatial scales.^5^ First, RNA within fixed cells is reverse-transcribed with tagged random hexamer primers to generate cDNA. FISSEQ uses the direct-ligation approach to produce cDNA fragments as templates for RCA, and these cDNAs produced from reverse transcription of mRNA are directly circularized using a single-stranded DNA ligase. Then, the cDNA fragments are circularized and amplified with RCA. The RCA amplicons are then cross-linked with BS(PEG)9, a bis-succinimide ester-activated PEG compound. BS(PEG)9 reduces the nonspecific binding of probes and has a highly fluorescent signal after the hybridization of the probe. This creates 200 to 400 nm DNA nanoballs, comprising tandem cDNA repeats of the target sequence, on top of a histology section. Partition sequencing using pre-extended sequencing primers with random mismatches at the ligation site reduces the total number of molecular sequencing reactions, resulting in a minimal signal-to-noise ratio or number of position changes after multiple rounds of rehybridized probing. In this manner, FISSEQ achieves sufficient spot density and RNA localization to discern individual molecules. FISSEQ uses color sequences at each pixel to identify objects. The putative nucleic acid sequences are determined for all pixels and compared with reference sequences. FISSEQ was used to confirm RNA expression and localization in human primary fibroblasts.^5^ The method can also examine other cell types, tissue sections, and whole-mount embryos for 3D visualization that spans multiple resolution scales.^56^ Single molecule detection is also possible, since FISSEQ improves optical resolution and reduces signal noise. But FISSEQ also has limitations; it is not suitable for all cellular structures and specific classes of RNA, for example detecting genes involved in RNA and protein processing.^56^ Nanotechnology elements supporting FISSEQ are DNA nanoballs, fluorescent probes, confocal imaging, and the cross-linking reagent BS(PEG)9.

#### 4.1.6 Spatially Resolved Transcript Amplicon Readout Mapping

STARmap uses targeted signal amplification and hydrogel–tissue chemistry interactions to enable 3D in situ transcriptomics in intact tissue (Figure 2C).^57^ A specific set of cellular RNAs are amplified in situ by a method called the Specific Amplification of Nucleic Acids via Intramolecular Ligation (SNAIL). SNAIL achieves high efficiency for in situ sequencing by avoiding a reverse transcription step. In SNAIL, two cDNA probes hybridize to the same RNA molecule. One of these probes (the padlock probe) contains a specific gene identifier. This probe is circularized, and RCA generates an amplicon in the form of a DNA nanoball that contains multiple copies of the specific gene identifier (Figure 2C).^57^ SNAIL provides a much higher absolute signal intensity and signal-to-noise ratio outcome than that obtained with smFISH probes. It also has much greater detection efficiency than single-cell RNA sequencing, despite having a simpler experimental procedure.^57^ After amplification, the DNA nanoballs are enzymatically modified and polymerized to form a hydrogel that serves as a 3D cDNA library (Figure 2C). Then, the RNA landscape is sequenced with the Sequencing with Error-Reduction by Dynamic Annealing and Ligation (SEDAL) process.^99^ SEDAL employs two kinds of short, degenerate probes: reading probes and fluorescence probes. The first kind decodes bases, and the second creates fluorescent puncta from the decoded sequences. These two probes bind DNA targets transiently and, after specific complementary ligation, form stable products for imaging with a confocal microscope. For multiplexed imaging, fluorescent signals are stripped with formamide, and another cycle of bases are read, eliminating the accumulation of errors during sequencing. STARmap was used to define cell types and activity-regulated gene expression in the mouse cortex, from mouse brain sections and larger 3D 150 mm-thick tissue blocks.^57^ A limitation of STARmap is that it cannot independently fully define brain cell typology in 3D anatomy. In the future, STARmap aims to study activity patterns exhibited or experienced by cells during behavior in real time. The nanotechnology tools that underlie STARmap include the SNAIL method, which incorporates DNA nanoballs; the SEDAL method, which incorporates fluorescent probes; and the confocal microscope for imaging.

#### 4.1.7 Slide-seq

Slide-seq is a spatial transcriptomics technology, in which DNA-barcoded beads are used to reveal spatial information about RNAs.^61,82,83^ In Slide-seq, DNA-barcoded, 10 μm beads are packed onto a rubber-coated glass coverslip to form a monolayer. RNAs from tissue sections are transferred onto the beads, with the precise locations of the beads preserving RNA spatial information. Then, the barcode sequence from each bead is determined by sequencing using oligonucleotide ligation and detection chemistry. The Slide-seq had low transcript detection sensitivity, limiting its utility. To address this limitation, researchers developed Slide-seqV2, an improved version of Slide-seq with an order-of-magnitude higher sensitivity that also had better methods for library generation, barcoded bead synthesis, and array sequencing. These modifications increased RNA capture efficiency to a level ∼10-fold greater than Slide-seq, a level approaching the detection efficiency of droplet-based single-cell RNA-seq techniques.^82^ The capture efficiency improvements within Slide-seqV2 make it useful across many experimental contexts. Nanotechnology methods important in Slide-seq are the use of beads 10 to 20 μm wide for location mapping, DNA barcodes, and the confocal microscope.

#### 4.1.8 Seq-Scope

Seq-Scope is a spatial transcriptomics method that relies on an array of randomly barcoded single-molecule oligonucleotides and two rounds of sequencing, conveniently achieved by the Illumina sequencing platform.^100^ Seq-Scope uses a array attached to a solid surface that contains single-stranded oligonucleotides, each containing a randomly generated barcode sequence called a High-Definition Map Coordinate Identifier (HDMI). The HDMI oligonucleotides are amplified, generating clusters, each with a specific HDMI sequence. In the first round of sequencing, each HDMI sequence and its spatial coordinates are determined by the Illumina platform. Then, HDMI clusters capture RNA released from an overlying tissue section and corresponding cDNA sequences are generated; these HDMI and cDNA sequences are determined in the second round of sequencing. In other words, the first round of sequencing provides the spatial information, and the second round of sequencing provides gene expression information. When the data from the two rounds of sequencing are combined, they allow construction of a spatial gene expression matrix. Seq-Scope has a spatial resolution of 500 to 800 nm (600 nm on average) and achieves submicrometer resolution, comparable to an optical microscope.^101^ Seq-Scope reveals the spatial transcriptome on multiple histological scales and has been used to distinguish tissues within an organ (eg, different regions of the liver and colon), different cell types, and different subcellular regions (eg, nucleus versus cytoplasm).^101^ Seq-Scope has several advantages, including high throughput, straightforward procedures, precise measurements, excellent breadth of transcriptome capture output, and high spatial resolution, making it far superior to most other technologies. Seq-Scope is limited, however, to the capture of the poly-A-tagged transcriptome, making it less robust than spatial-CITE-seq or DBiT-Seq, which are capable of spatially profiling the transcriptome alongside protein expression. The nanotechnology supporting Seq-Scope is the set of HDMI barcode sequences.

### 4.2 Spatial Proteomics

Spatial proteomics, facilitated by nanotechnology, has revolutionized our understanding of cellular organization and function at the molecular level (Figure 1). By employing nanosized materials and techniques, researchers can precisely map the spatial distribution of proteins within cells, tissues, and organs, unlocking insights into complex biological processes with in more detail and better resolution.^102^ The synergy between nanotechnology and proteomics has been achieved by integrating high-end imaging techniques, such as LCM microscopy, Multiplexed Ion Beam Imaging (MIBI), or CO-Detection by IndEXing (CODEX). Sample processing techniques, such as Expansion Proteomics (ProteomEx)^103^ or One Pot for Trace Samples (nanoPOTS),^104^ have enabled the capture of nanoscale specimen volumes for multiplexed mass spectrometry. Additionally, streamlined spatial workflows, such as Single-Cell Deep Visual Proteomics (scDVP)^105^ or 3D imaging of Solvent-Cleared Organs Profiled by Mass Spectrometry (DISCO-MS),^106^ allow for the powerful and unbiased characterization of biological heterogeneity. These spatial proteomics tools are described below.

#### 4.2.1 Laser Capture Microdissection Microscopy

The imaging technique LCM microscopy has played a pivotal role in understanding cellular heterogeneity with nanoscale precision, offering the ability to study specific subcellular regions of interest, facilitating in-depth examination of protein distributions and interactions.^107^ In general, LCM enables the targeted dissection of individual cells or subcellular structures from complex biological samples, which are then viewed with a microscope. And in spatial proteomics specifically, LCM is instrumental for analyzing protein distribution within cellular compartments, studying protein interactions, and unraveling signaling pathways in the cellular microenvironments.^108^ By precisely isolating organelles from an otherwise complex heterogeneous tissue section, researchers can analyze their proteome composition, providing insight into their function and dynamics in local cell populations without losing spatial information.

Individually tailored therapies, guided by the molecular profiling of biopsy samples, have traditionally relied on techniques such as immunohistochemistry and bulk genomic analysis.^109^ While analyzing whole tissue specimens has shown promise in predicting patient responses to chemotherapy, the process of extracting these specimens introduces significant variability,^110^ which stems from the diverse cellular composition of tissues, the uncertainty surrounding the proportion of tumor versus host cells in the sample, and the loss of spatial information about cell types within the tissue. Hence, LCM has emerged as an ideal technology to dissect cells at nanoscale for tissue spatial profiling to allow for proteomic analysis of specific cells or cell subsets while preserving their spatial context.

Given its ability to dissect nanometer regions, LCM has been paramount for understanding the spatial organization of tumor and immune cell populations in tumor immunology and subclonal analysis, offering invaluable insights into immunotherapy responses and the emergence of drug resistance.^108,111^ Combining LCM with certain other nanotechnologies, such as Cytometry by Time-of-Flight (CyToF)^112^ and the NanoString nCounter gene expression system,^113,114^ has offered analysis of post-translational modifications and their functions in signaling pathways. LCM-guided mass spectrometry methods are rapidly advancing for discovery applications from region-of-interest to single-cell resolution; and mass spectrometry experts are beginning to realize the dream of robust, high-yield LCM single-cell tissue proteomics from either the same thin-tissue section or precision-registered serial sections from a variety of tissue types. LCM microscopy also offers high-yield single-cell transfer to a nanochip, subcellular precision, and high throughput.

Despite its potential, LCM microscopy has its drawbacks, including the time-consuming nature of several steps: visualization, manual cell selection, and collection processes. Typically, the choice of cells for LCM analysis is made through direct microscopic observation, but this approach is sometimes hindered by the poor image quality resulting from the necessity of keeping tissues uncovered during the process. But advancements in digital imaging, liquid coverslip chemistry, artificial intelligence, and automation are anticipated to overcome these challenges and revolutionize the field of tissue spatial profiling in the future.^107^

#### 4.2.2 Multiplexed Ion Beam Imaging

**Figure 3:**
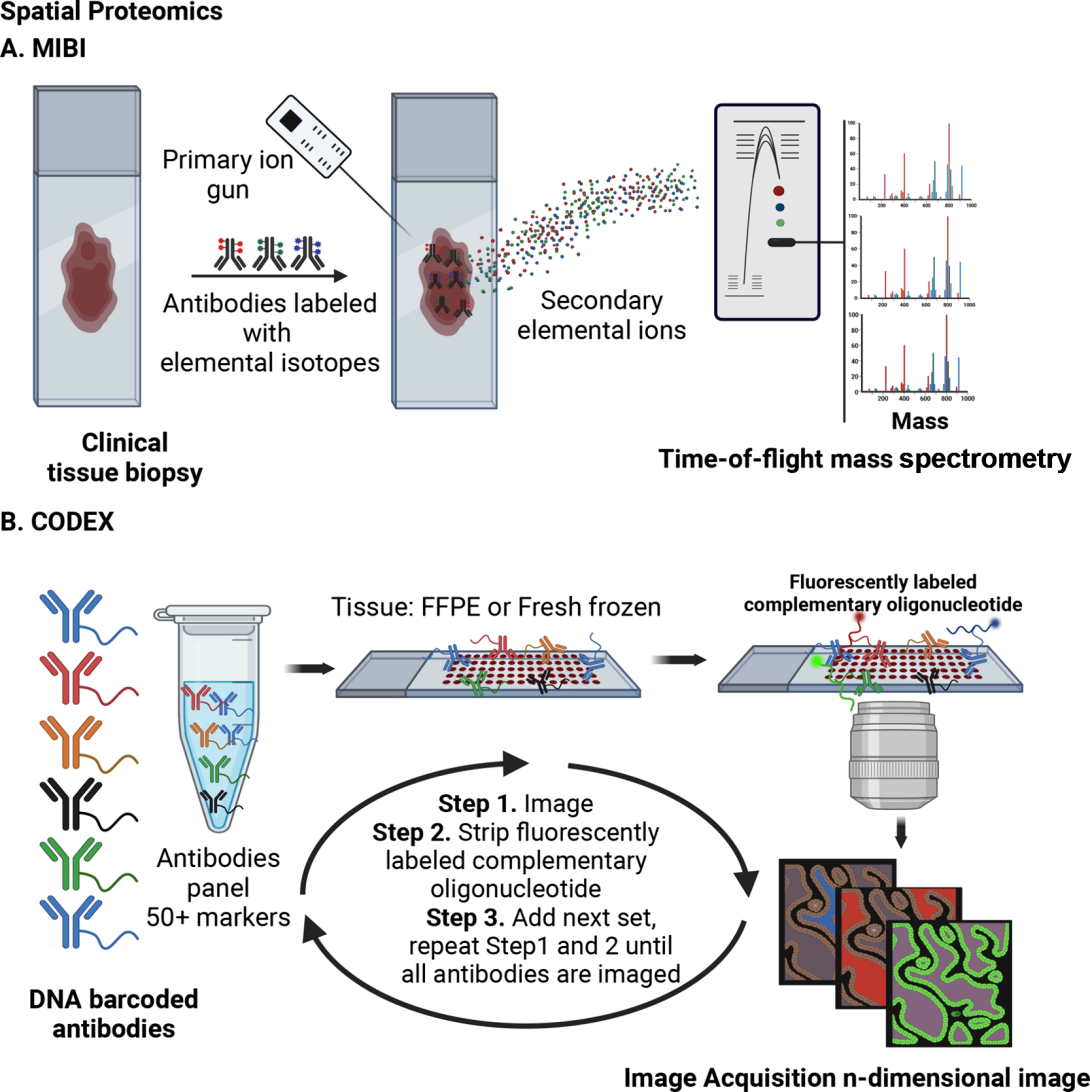
Spatial proteomics methods. A. MIBI workflow.^119^ FFPE samples are exposed to a panel of antibodies labeled with metal isotopes. An ion beam rasters across the tissue grid, liberating ions, including from the isotope labels bound to proteins in the tissue via specific antibodies. Time-of-flight mass spectrometry separates the labels based on mass for the detection of proteins present in the tissue. B. CODEX workflow.^123,124^ FFPE or fresh frozen tissue is exposed to a panel of antibodies, each conjugated with a specific oligonucleotide (DNA) barcode. The tissue is then stained with three complementary oligonucleotides conjugated to fluorescent dyes. After imaging, the first set of oligonucleotides is stripped off, another set of oligonucleotides conjugated to fluorescent dyes is added, and the tissue is imaged again. This cycle is repeated until all antibodies from the panel have been imaged. Part A was adapted with permission form ref (119). Copyright 2020 Springer Nature. Part B was adapted with permission under a Creative Commons CC-BY license form ref (124). Copyright 2018, published by Elsevier. The figures were created with BioRender. Abbreviations: MIBI: Multiplexed Ion Beam Imaging; FFPE: Formalin-Fixed Paraffin-Embedded; CODEX: CO-Detection by Indexing.

Like LCM microscopy, MIBI has also helped to understand cellular heterogeneity with nanoscale precision, allowing for the in-depth study of specific subcellular regions of interest and proteins.^115,116,107,117^ In MIBI, the tissue is first stained with a set of antibodies labeled with metal isotopes (Figure 3A). An ion beam rasters across the tissue, liberating ions that feed into a Time-of-Flight Secondary Ion Mass Spectrometer (ToF-SIMS), which separates the labels by mass (Figure 3A). Knowing which isotope label is bound to which antibody, researchers can determine which target proteins are present, and because the ion beam is rastered across the tissue, multiplex images can be created.^118,119^ To titrate the optimal concentration of antibodies for MIBI, labeled antibodies are screened in tissue microarrays. ToF-MS is utilized to separate the marker labels for identification within the original tissue. These images are partitioned to define cell–cell boundaries, which allow cell phenotypes to be described as distances between signals.

MIBI has been applied to study the tumor microenvironment, identifying cell phenotypes and analyzing spatial relationships across numerous tumor types, such as the spatial relationships between immune and cancer cells and the specific locations of immunoregulatory proteins.^118−121^ Advantages of MIBI center around its high-parameter capabilities, high sensitivity, and subcellular resolution. Recent advances for MIBI using ion beam tuning targets image resolution at varying depths via multiple *z*-direction scans, allowing for reconstruction of 250 nm 3D images in the axial direction.^122^ But MIBI also has drawbacks: long imaging times and high cost. The processing time of mass spectrometry data obtained from each pixel and converting it into derivative spatial images also confines the sample area.^4^

Nanotechnology used in MIBI includes staining with metal-labeled antibodies and data acquisition with the ToF-SIMS. A related nanotechnology is the MIBIscope, a dynamic ToF-SIMS instrument that uses a gold liquid metal ion gun as its primary ion source and produces a live image of tissue topography using a secondary electron detector.^119^ The results of this study illustrate that MIBI, using MIBIscope, achieves high sensitivity and resolution when studying the spatial tumor immune landscape.

#### 4.2.3 CO-Detection by IndEXing

CODEX is a multiplexed single-cell imaging technology that uses DNA-barcoded antibodies for spatial proteomics (Figure 3B). In CODEX, target proteins in <10 μm-thick FFPE or fresh frozen tissue sections are labeled with a large panel of antibodies, each conjugated to a specific oligonucleotide barcode (Figure 3B). These barcodes are detected, three at a time, in several rounds of hybridization and imaging. In each round, complementary oligonucleotides, each labeled with a fluorescent dye, bind to the barcodes, and the tissue is imaged. Then, a gentle washing step removes the fluorescent dyes. This process is repeated until all the barcodes—and the protein targets they represent—are detected.^123^ CODEX employs a cyclic fluidic device to automate the rounds of hybridization and imaging,^2^ and it can be integrated with any tricolor epifluorescence microscope (Figure 3B). CODEX has been used for cancer, autoimmunity, and infection research.^123^ It is capable of spatial resolution around 260 nm in the lateral (xy) and axial (z) dimensions to create 3D images.^124^ But CODEX requires special reagents and equipment and has several challenges due to it being a fluorescence-based multiplexed imaging technology,^123^ including limitations associated with the microscope system, background autofluorescence, and the rapid processing of large-scale imaging data sets. Nanotechnology elements supporting CODEX include the microfluidics system for repeated target probing, DNA-barcoded antibodies, and the tricolor fluorescence microscope, such as the Keyence BZ-X710 fluorescence microscope configured with 3 fluorescent channels (FITC, Cy3, Cy5).

Current antibody-based spatial proteomics methods have some general limitations. First, the number of protein targets is limited. MIBI can image up to 100 targets simultaneously after performing SIMS, although commercially available products are only capable of detecting around 40 targets.^119^ The CODEX workflow visualizes 50+ protein targets at the single-cell level,^124^ and the updated CODEX multiplexed imaging platform can detect 100 RNA labels.^122^ Second, antibody detection methods are subject to nonspecific binding, epitope loss, and tissue degradation. Additional limitations for antibody methods are related to the size of the capture region of interest within the tissue slide, the time needed for fluorescent image acquisition, and the cost of mass spectrometry detection. Furthermore, these methods are based on relative spectral intensities and are only semiquantitative.^1−3^

#### 4.2.4 Additional Techniques for Sample Processing and Streamlined Spatial Workflow

Nanotechnology plays a crucial role in proteomics based on mass spectrometry, spanning various applications and workflows. From sample pretreatment to mass spectrometry analysis, nanoscale processing is integral, especially in single-cell analysis (a cornerstone in several applications in the biomedical field). While conventional proteomic methods based on mass spectrometry require samples comprising more than thousands of cells to profile in-depth identification, innovative platforms such as nanoPOTS offer enhanced recovery and efficiency by minimizing sample volumes to less than 200 nL, allowing for the identification of ∼1500 to ∼3000 proteins from ∼10 to ∼140 cells, respectively.^125^ Despite advancements in imaging-based and MS-based methods, integrating nanotechnology remains a challenge, particularly in connecting imaging data with protein abundance measurements that have single-cell resolution. One platform, scDVP, offers a promising solution by combining three techniques: cellular phenotype image analysis driven by artificial intelligence, automated single-nucleus and single-cell LCM, and ultrahigh-sensitivity mass spectrometry coupled with a nanoelectrospray ion source. DVP enables the discovery and characterization of cellular interactions and states with the added advantage of analyzing the subcellular structures and spatial dynamics of extracellular matrix.^126^

Spatial molecular profiling of complex tissues is further enhanced by nanoscale staining techniques. For instance, DISCO-MS combines whole organism clearing, image analysis based on deep learning, robotic tissue extraction assisted by nanoboosters, and ultrahigh-sensitivity mass spectrometry to yield proteome data identifying more than 6,000 proteins across various clearing conditions.^127^ Other nanotechnologies combined with mass spectrometry to allow high resolution spatial profiling include ProteomEx, which—using manual microsampling without custom or special equipment—enabled quantitative profiling of the spatial variability of the proteome at ∼160 μm lateral resolution in mammalian tissues, equivalent to the tissue volume of 0.61 nL.^128^ In addition, a Microscaffold Assisted Spatial Proteomics (MASP) strategy, based on spatially resolved microcompartmentalization of tissue using a 3D-printed microscaffold, mapped more than 5000 cerebral proteins in the mouse brain, including numerous important brain markers, transporters, and regulators, that were identified by a trapping nano-LC and high-resolution mass spectrometry system.^129^ Furthermore, Mass Spectrometry Imaging (MSI) is another powerful tool for mapping of the spatial distribution of proteins by label-free quantification in biological tissues. For instance, Nanospray Desorption Electrospray Ionization (nano-DESI) MSI generates multiply charged protein ions, advantageous for the identification of top-down proteomics analysis, achieving proteoform mapping in mouse tissues with a spatial resolution down to 7 μm.^130^

### 4.3 Spatial Metabolomics

Developed only two decades ago, spatial metabolomics is another emerging field within spatial omics that has enabled the identification of metabolites within the spatial contexts of cells, tissues, organs, and organisms.^131^ Spatial metabolomics uses imaging technology based on mass spectrometry, including Matrix-Assisted Laser Desorption/Ionization (MALDI) MSI,^3,132^ Desorption Electrospray Ionization (DESI) MSI,^133,134^ and SIMS imaging.^135^

#### 4.3.1 Matrix-Assisted Laser Desorption/Ionization-Mass Spectrometry Imaging

**Figure 4:**
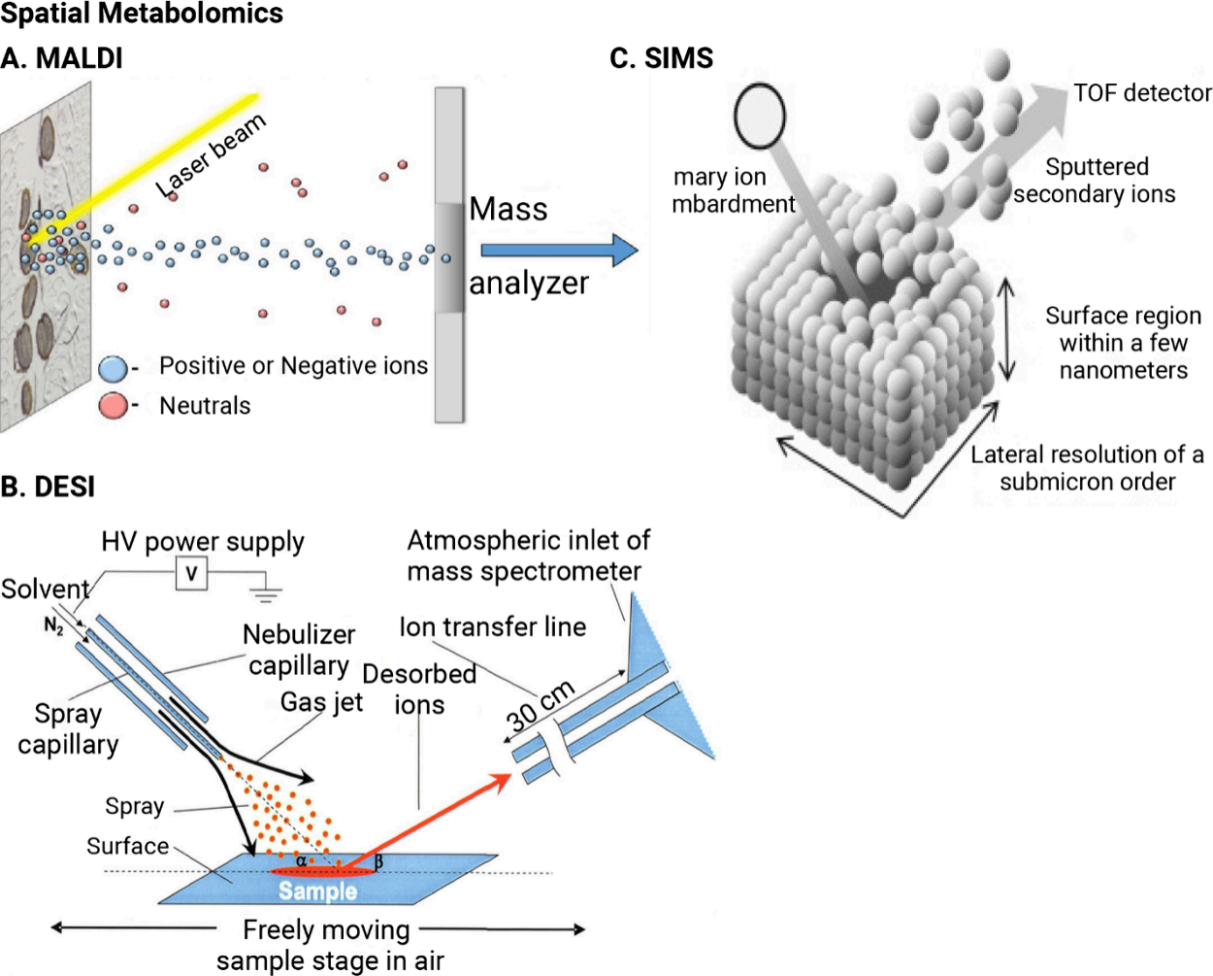
Spatial metabolomics methods. Each technique employs frozen or FFPE tissues,^133^ and processes before MSI are shown. A. Schematic of MALDI. MALDI requires a sample preparation step; tissue samples are first coated with a low molecular weight matrix.^140,167^ B. Schematic of DESI.^168^ DESI directly sprays samples with an electronically charged solution for ionization, allowing desorption via a solvent stream under ambient conditions.^147,168^ C. Schematic of SIMS. SIMS bombards sample surfaces with an ion beam to induce ionization and desorption in an ultrahigh vacuum.^33,58^ Part A was reproduced with permission from ref (167). Copyright 2016 Elsevier and the Copyright Clearance Center. Part B was reproduced with permission from ref (168). Copyright 2004 The American Association for the Advancement of Science. Part C was reproduced with permission ref (58). Copyright 2016 Elsevier and the Copyright Clearance Center. Abbreviations: FFPE: Formalin-Fixed Paraffin-Embedded; MSI: Mass Spectrometry Imaging; MALDI: Matrix-Assisted Laser Desorption/Ionization; DESI: Desorption Electrospray Ionization; SIMS: Secondary Ion Mass Spectrometry Imaging.

MALDI is an ionization method used in conjunction with MSI for spatial metabolomics. MALDI requires a sample preparation step that involves mixing the sample with a protective low molecular weight matrix before spotting the mixture onto stainless steel and allowing it to crystallize. Next, the samples are exposed to a scanning laser, transforming solid components into charged gaseous particles, ionizing the sample within a 10 μm-wide window (Figure 4A). Finally, mass spectrometry detects these ions to define each metabolite image location (Figure 4A).^136^

MALDI mass spectrometry imaging has benefits, but also limitations. It has better metabolite coverage than other spatial metabolomics methods, and consistently detects hundreds of metabolites at a spatial resolution of around 10 μm.^133,136,137^ Even better, atmospheric pressure-MALDI developed by Spengler’s group achieves a spatial resolution of 1.4 μm,^138^ yet the resolution is still worse than the spatially resolved mass spectrometry approaches used for spatial proteomics.^132,139^ The resolution often suffers when these approaches are applied to metabolomics, to accommodate mass spectrometry instrument sensitivity to low-abundance species from small areas. Spatial resolution and sensitivity are inherently connected in spatial metabolomics techniques; as the diameter of the laser spot decreases to achieve a finer spatial resolution, the ion yield usually decreases as well.^136,137^ Thus, researchers struggle to achieve finer spatial resolution while maintaining adequate signal intensities. Other limitations of MALDI-MSI include decreased resolution caused by delocation (when molecules diffuse across or away from the tissue) and difficulty detecting low-weight molecules (<600 Da). The matrix ions may have similar profiles with multiple lower-weight metabolite ions, which can interfere with the visualizations of select metabolites, defining a low-weight molecule detection problem.^140,141^

Although mass spectrometry is the primary nanotechnology tool in MALDI-MSI, nanomaterials have been used as alternative matrices to improve various aspects of the method. Some researchers have increased its sensitivity by adding nanoparticles to the low-density matrices, which homogeneously concentrates targets into a narrow ring, similar to the characteristic ring-like pattern observed after a drop of spilled coffee evaporates (the “coffee ring effect”). Advantages of sample concentrating using this method led to higher signals relative to conventional MALDI, especially for analytes with greater mass-to-charge ratios.^142^ Other researchers used nanoparticles to enhance detection of triacylglycerols from lipid mixtures, which are overwhelmed by other lipids in conventional MALDI detection. They found a matrix containing citrate-capped gold nanoparticles enhanced the cationization of triacylglycerols and effectively suppressed other lipid signals, aiding triacylglycerol detection.^143^ And in glycomics studies, MALDI matrices containing graphene nanosheets and carbon nanoparticles improved sensitivity in the detection of native glycans, which ionize inefficiently.^144^

#### 4.3.2 Desorption Electrospray Ionization Mass Spectrometry Imaging

DESI is another ionization method used in conjunction with MSI for spatial metabolomics.^145,146^ DESI directly sprays samples with an electronically charged solution for ionization, allowing desorption via a solvent stream under ambient conditions (Figure 4B).^147^ As the DESI ionization probe scans across the tissue sample, desorbed ions from the tissue enter the mass spectrometer, which collects mass-to-charge ratio information that can be correlated with the spatial distribution (Figure 4B).^148^ Unlike MALDI-MSI, which requires a sample preparation step, DESI-MSI can provide spatial information about metabolites with little to no sample preparation and does not need a matrix. It also does not suffer from spatial assignment errors caused by sample movement.^147,149^ But limited spatial resolution is a major challenge for DESI-MSI. Most studies have reported spatial resolutions of only 50 to 200 μm due to multiple factors such as solvent composition, capillary size, and gas flow rate.^150^ And in addition to these factors, resolution is also limited when balancing sensitivity for low abundance species, as in MALDI-MSI. To improve the resolution, Laskin et al. developed nano-DESI MSI, which used two fused silica capillaries: a primary capillary that supplied solvent and maintained a liquid bridge with the sample, and a secondary capillary that transported the analyze to the mass spectrometer.^35,151^ Next, they developed an approach to control the distance between the nano-DESI probe and the sample with shear force microscopy for MSI in constant-distance mode, thereby achieving ∼11 μm spatial resolution in images of mouse pancreatic islets.^152^ The researchers also coupled a portable nano-DESI probe to a drift tube ion mobility spectrometry-mass spectrometer, which allowed imaging of drift time-separated ions of mice uterine tissues with a spatial resolution less than 25 μm.^153^ An ion mobility spectrometer recorded the drift time to determine the ion mobility.^154^ An ion mobility spectrometer recorded the drift time, meaning the time it takes for each ion to reach a detector. In addition to its resolution issues, another challenge of DESI-MSI is its sensitivity and specificity. This has been improved by adding silver ions to the nano-DESI solvent, but only for analytes containing double bonds.^155^ Nanotechnology tools that support DESI-MSI include MSI, nanospray (ie, nano droplets), and the DESI ionization probe.

#### 4.3.3 Secondary Ion Mass Spectrometry Imaging

SIMS is yet another ionization method used in conjunction with MSI for spatial metabolomics. Rather than a laser or charged spray, a primary ion beam scans across the sample, bombarding the surface to induce ionization in an ultrahigh vacuum (Figure 4C).^33^ The ionization of molecules at the sample surface generates a secondary beam of sputtered ions of opposite polarity, which are transferred to a mass analyzer (Figure 4C).^156^ An advantage of this method is spatial resolution. The primary ion beam is highly focused and impacts samples with an orthogonal angle, as opposed to the oblique angle utilized for desorption catalysts in MALDI and DESI. This degree of control enhances spatial resolution, which can reach as low as 50 nm,^157^ making it possible to distinguish molecules between different organelles of the same cell.^158^ But the high energy ion beam (1 to 70 keV) is highly destructive, leading to the fragmentation of biomolecules during desorption. As such, types of SIMS combining high energy beams with high dose density (ie, > 10^13^ ion/cm^2^ as in dynamic SIMS) can only target monatomic or diatomic elements, limiting their application in spatial metabolomics.^159^ Types of SIMS employing ion beams of decreased dose density (ie, static SIMS) still produce degradation, but to lesser extent, and initial versions of these methods provided sufficient resolution to quantify biomolecules up to 300 Da.^160^ To improve static SIMS, researchers have modulated the primary ion beam to decrease sample destruction and increase ionization efficiency, allowing for increased sensitivity to detect biomolecules of lower concentration and higher molecular weight. Metal cluster ion beams, composed of Au^3+^ or Bi^3+^, expanded the ability of SIMS to analyze low molecular weight biomolecules such as metabolites and lipids,^161^ while small cluster ion beams, composed of C_60_ for example, enabled the analysis of high molecular weight biomolecules such as peptides and proteins.^162^ The range of mass resolution was further increased by the introduction of gas cluster ion beams, which improved the ionization efficiency of fully intact biomolecules up to 100-fold compared to that achieved by metal or small cluster ion beams.^163^ Despite these advances in mass resolution and dynamic range, the diminished dose density of static SIMS increases dispersion of the primary ion beam, decreasing the spatial resolution to a range of 550 to 900 nm.^164,165^ Nanotechnology tools in SIMS imaging include mass spectrometry, ion beams, and the nanoparticle coating employed to enhance ionization efficiency in metal-assisted SIMS.^166^

### 4.4 Spatial Epigenomics

Epigenetic modifications (to histones or DNA) control the state of chromatin, affecting DNA accessibility; open chromatin allows gene expression to occur, while closed chromatin prevents gene expression. Thus, these reversible epigenetic modifications affect cellular function and explain biological phenomena on the cellular level (Figure 1). Spatial epigenomics provides information about epigenetic modifications across a population of cells or across a tissue, revealing global epigenetic changes. Spatial epigenomics methods include spatial-ATAC-seq and spatial-CUT&Tag.

#### 4.4.1 Spatial Assay for Transposase-Accessible Chromatin and RNA Using Sequencing

Based on DBiT-seq, spatial-ATAC-seq is a method that provides a genome-wide map of open and accessible chromatin regions in intact tissue sections.^156^ Spatial-ATAC-seq utilizes the in situ Tn5 transposition chemistry^169^ and microfluidic deterministic barcoding as described in DBiT (see section 4.5.2).^54^ Spatial-ATAC-seq employs the Tn5 transposon to insert DNA oligomers into genome accessible locations on fixed sections,^170,171^ and adapters containing a ligation linker are added to label the modified genome accessible sites. Next, a grid of barcodes is overlaid using microchannels, and these location coordinate markers are ligated to the Tn5-generated oligos in successive rounds, creating a map of barcode combinations. The array of barcodes is then imaged and overlaid onto tissue morphology, revealing the locations of accessible chromatin. Then, reverse cross-linking frees barcoded DNA fragments, creating a 2,500-tile spatial tissue mosaic, which is amplified by Polymerase Chain Reaction (PCR) and is the input for preparation of sequencing libraries. Spatial-ATAC-seq has the ability to capture spatial epigenetic information within the mouse and human brain.^156^ And the method has also been applied to mouse embryos to delineate the epigenetic landscape of organogenesis, and in human tonsils to map the epigenetic state of different immune cells.^156^ Advantages of the method are high spatial resolution, high yield, a high signal-to-noise ratio, and a pixel size of 20 μm at the cellular level.^156^ A disadvantage of spatial-ATAC-seq is that, unlike single-cell technologies, detected pixels may contain partial nuclei or multiple nuclei, and thus signals may comprise multiple cell types, which complicates data interpretation. The nanotechnology underlying spatial-ATAC-seq is microfluidic deterministic barcoding.

#### 4.4.2 Spatial Cleavage Under Targets and Tagmentation

**Figure 5:**
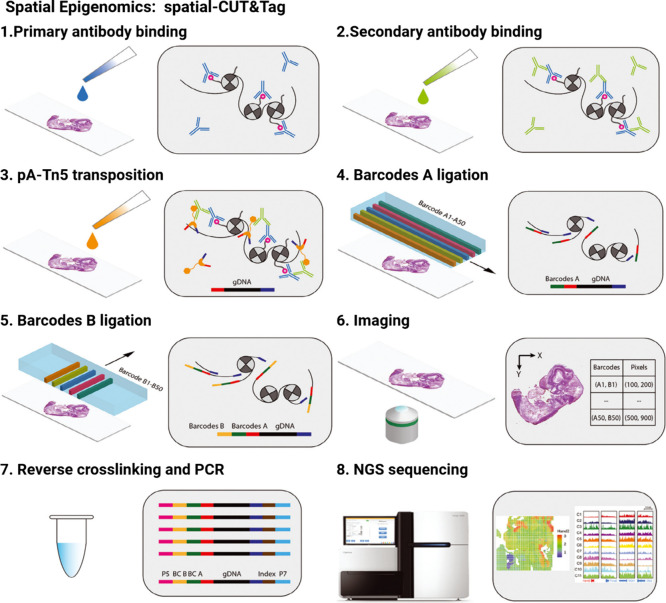
Spatial epigenomics method spatial-CUT&Tag. Schematic of spatial-CUT&Tag workflow.^175^ Primary antibodies that bind histone modification sites are added to fixed tissue followed by secondary antibodies. The next step involves pA-Tn5 directed transposition into DNA. Then, two sets of barcodes (A and B), delivered by microchannels, are flowed over the tissue surface. Ligation of these barcodes creates a 2D labeling grid for imaging. The final step is reverse cross-linking and PCR followed by next-generation sequencing. The figure was reproduced from ref (175). Copyright 2022 The American Association for the Advancement of Science. Abbreviations: gDNA: genomic DNA; 2D: two-dimensional.

Spatial-CUT&Tag analyzes single-cell epigenomes by profiling chromatin states in situ within tissue sections, and achieves an unbiased, genome-wide epigenomic map (Figure 5). The approach is based on in situ microfluidic deterministic barcoding,^54,172^ Cleavage Under Targets and Tagmentation (CUT&Tag) chemistry,^173,174^ and next-generation sequencing. In the first step of spatial-CUT&Tag, antibodies that bind histone modification sites are added to the tissue, followed by secondary antibodies that tether a pA-Tn5 transposome (a form of fusion enzyme used for CUT&Tag) (Figure 5). The transposome complex is then activated, ligating linkers and insertions into genomic sites adjacent to specific histone marks defined by the primary antibodies (Figure 5).^175^ As in DBiT-seq and spatial-ATAC-seq, two sets of barcodes (A and B), delivered by microchannels, are flowed over the tissue surface (Figure 5).^54,156,172^ Ligation of these barcodes creates a 2D labeling grid, which is then imaged to link the tissue morphology to the spatial epigenomics map. The output assay signal is released after cross-link reversal, producing a library for sequencing quantitation (Figure 5).^175^ Spatial-CUT&Tag defined histone modifications within the cortical layer of mouse brain during development, highlighting the spatial patterning of cell types.^175^ Despite this utility, the method has resolution limitations, with a current spatial resolution of 20 μm pixels. To achieve higher precision in spatial multiomics profiling, one could combine reagents of DBiT-seq and spatial-CUT&Tag for microfluidic in-tissue barcoding.^156^ A serpentine microfluidic channel or increasing the number of barcodes could also help, reducing pixel size within the epigenome mapping area. Using these two methods, Fan et al. achieved simultaneous epigenomic and transcriptomic profiling on tissues from embryonic and juvenile mouse brain and from adult human brain with near–single-cell resolution.^176^ The epigenome was evaluated using spatial-CUT&Tag–RNA-seq applied to histone modifications, and mRNA expression was determined using spatial-ATAC–RNA-seq Spatial epigenome–transcriptome cosequencing overlays spatial multiomics signals, synergizing data from each method and allowing for the examination of mechanistic relationships across the central dogma of molecular biology. The nanotechnology supporting spatial-CUT&Tag is in-tissue microfluidic deterministic barcoding.

### 4.5 Spatial Multiomics

Spatial multiomics tools combine detection of distinct biomolecular domains inside an overlapping assay window and are the goal for the field. Vickovic and Lötstedt developed and published a spatial multiomics platform in 2022.^53^ Their automated and high-throughput approach mapped regional RNA expression via sequencing-based biomarkers and overlaid protein signals via DNA-barcoded antibodies or immunofluorescence labels. This approach enabled the simultaneous spatial evaluation of 96 sequencing-ready RNA libraries and 64 in situ protein targets in 2 days.^53^ Another spatial multiomics strategy uses the GeoMx Digital Spatial Profiler (DSP) from NanoString. Unlike the spatial multiomics platform developed by Vickovic and Lötstedt, which is limited to frozen tissue, the GeoMx DSP platform can be used on FFPE tissue sections. And it is capable of spatial analysis profiling for the whole transcriptome (18,000 RNA targets) and more than 96 proteins simultaneously. The GeoMx DSP, DBiT-seq, spatial-CITE-seq, and MOSAICA are exciting methods that query spatial RNA and protein expression.

#### 4.5.1 GeoMx Digital Spatial Profiler

**Figure 6:**
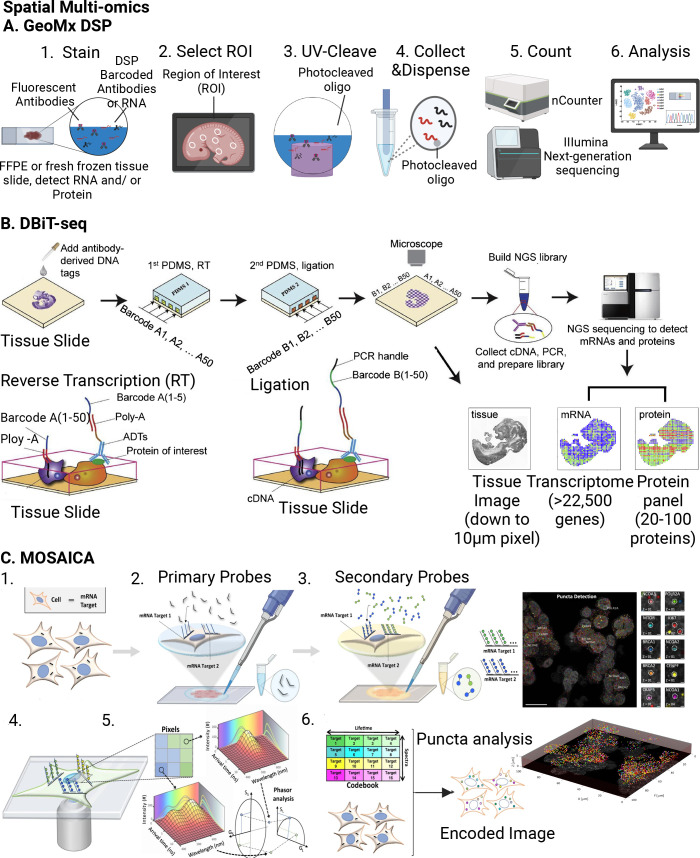
Spatial multiomics methods. A. The workflow for GeoMx DSP.^177,178^ Proteins and/or RNAs in tissue are labeled with oligos and regions of interest are selected. Oligos are cleaved with UV light, collected, and counting using the nCounter or next-generation sequencing before the final computational analyses. B. The workflow for the DBiT-seq platform.^54^ ADTs are exposed to tissue slides for protein detection. Then, a PDMS microfluidic chip with parallel channels is placed directly against the tissue slide. Fifty parallel microfluidic channels in the chip deliver a set of barcodes (set A) and RT to the tissue. Then another PDMS chip is placed on the tissue slide, containing channels that deliver another set of barcodes (set B) and DNA ligase to attach the B barcodes to the A barcodes, creating a 2D mosaic of tissue pixels. Then the cDNA is collected and amplified, and proteins and mRNAs are detected by next-generation sequencing. C. Schematic of the MOSAICA approach.^55^ The tissue is incubated with a set of primary 25- to 30-base nucleic acid probes that tile a specific mRNA, binding to complementary regions and containing adapters. Then, for detection, the samples are incubated with the secondary probe set containing pairs of fluorophores that hone to adapters on the primary set of probes. The resulting probe map can be imaged via a fluorescent microscope to capture the collection of spectral readouts and temporal lifetime features. Refining these raw data, bioinformatics-based tools direct the reconstruction of images by spectral and fluorescence lifetime signal processing. These images combine numerous target confocal detections, providing transcript levels, localization within a 3D reconstructed image, and are overlaid onto microscopic tissue structures. Part A was created with BioRender. Part B was reproduced with permission from ref (54). Copyright 2020, Elsevier. Part C was reproduced with permission under a Creative Commons CC-BY license from ref (55). Copyright 2022, published by Springer Nature. Abbreviations: DSP: Digital Spatial Profiler; oligos: oligonucleotides; UV: Ultraviolet; PDMS: Polydimethylsiloxane; ADT: Antibody-Derived DNA Tag; RT: Reverse Transcriptase; DBiT-seq: Deterministic Barcoding in Tissue for Spatial Omics Sequencing; MOSAICA: Multi-Omic Single-Scan Assay with Integrated Combinatorial Analysis; 2D: two-dimensional; 3D: three-dimensional.

The GeoMx DSP currently enables detection and imaging of RNA or protein on either FFPE or fresh frozen whole tissue sections. The workflow starts with staining of the prepared tissue (a 5 μm-thick section) with antibodies and/or RNA attached to oligonucleotide tags (ie, barcodes) via light-sensitive linkers (Figure 6A). Next, the GeoMx DSP automated microscope is used to select regions of interest (in a varying size of 10 to 600 μm in diameter). From the regions of interest, the microscope uses UV light to cleave the oligonucleotide tags and collects the oligonucleotides (Figure 6A). Then, the oligonucleotides are analyzed with the NanoString nCounter System to quantify levels of specific proteins or RNAs (Figure 6A). Finally, data visualization and analysis are performed.^177,178^ Since the instrument was launched in March 2019, many groups have utilized the GeoMx DSP to study biomolecular expression in carcinomas, supporting its use as a standard tool for oncology research. The use of equivalently tagged oligonucleotides allows the system to interrogate numerous RNA and protein biomarkers with higher throughput, and GeoMx DSP simultaneously profiled six nodular and six infiltrative cancer samples, interrogating 1812 RNA targets.^179^ One study combining Single-Cell RNA Sequencing (scRNA-seq) transcriptomes and spatial transcriptomics identified Activin A as a paracrine-acting factor that contributed to tumor progression.^75^ A current limitation of the GeoMx DSP is its inability to achieve single-cell resolution for biomarker coexpression due to low protein detection efficiency.^78,179^ Nanotechnology tools that underscore GeoMx DSP include RNA probes conjugated to fluorophores to interrogate specific biomolecule targets, the imaging platform, and oligonucleotide barcodes.

#### 4.5.2 Deterministic Barcoding in Tissue for Spatial Omics Sequencing

DBiT-seq is a microfluidics-based platform for analyzing spatial proteomics and transcriptomics, created by Fan et al.^54,180^ In DBiT-seq, tissue sections are exposed to Antibody-Derived DNA Tags (ADTs) for protein detection. For RNA detection and spatial analysis, a Polydimethylsiloxane (PDMS) microfluidic chip is placed directly against the tissue slide (Figure 6B). Fifty parallel microfluidic channels in the chip deliver a set of oligo-dT-tagged DNA barcodes (set A), along with reverse transcriptase into lanes on the surface of the tissue slide. Then another PDMS chip is placed on the tissue slide, containing channels that deliver another set of oligo-dT-tagged DNA barcodes (set B), along with DNA ligase to attach the B barcodes to the A barcodes, creating a 2D mosaic of tissue pixels (Figure 6B). After imaging by a microscope to define histological features, the cDNA is collected and amplified to build a next-generation sequencing library. Finally, proteins and mRNAs are detected by next-generation sequencing (Figure 6B).^54^ DBiT-seq has been applied to study mouse embryos to measure a panel of 22 proteins and mRNA transcriptome.^54^ It has also been used for transcriptome sequencing within embryonic and adult FFPE sections, at cellular resolution (25 μm pixels) and >1000 gene per pixel coverage.^172^ Performing spatial whole transcriptome sequencing on FFPE samples without tissue dissociation or RNA exaction is one of the strengths of DBiT-seq as an in-tissue barcoding approach. A weakness is that, even though the pixel size of DBiT-seq can be scaled down to 10 μm, it is still not capable of directly resolving single-cell spatial mapping. Nanotechnologies exemplified in DBiT include antibody-derived DNA tags for protein detection, subnanometer microfluidic chambers for creating a spatial barcoding grid, and the optical or fluorescence microscope for detection.

#### 4.5.3 Spatial Co-Indexing of Transcriptomes and Epitopes for Multi-Omics Mapping by Highly Parallel Sequencing

Spatial-CITE-seq extends Coindexing of Transcriptomes and Epitopes (CITE-seq) to the spatial dimension and enables multiplexed protein and whole transcriptome comapping.^181^ The first step of this method uses a cocktail of ∼200–300 ADTs to stain a paraformaldehyde-fixed tissue section. The ADTs include a poly(A) tail, a Unique Molecular Identifier (UMI) tag, and a DNA sequence that is specific for select antibodies.^181,182^ As in DBiT, two sets of barcodes (A, row and B, column) are introduced using different microfluidic chips for ligation in situ, creating a 2D grid of tissue pixels to coindex all the protein epitopes and the transcriptome. The collection of barcoded cDNAs is then amplified by PCR and used for next-generation sequencing library preparation for paired-end sequencing of both ADTs and cDNAs, allowing the spatial reconstruction of protein and RNA coordinates. Spatial-CITE-seq incorporates 200 to 300 protein markers, substantially enhancing tissue mapping at cellular resolution, and offers the highest multiplexing to date for spatial protein profiling. Spatial-CITE-seq can profile 189 proteins and whole transcriptomes in multiple mouse tissue types and 273 proteins and the whole transcriptome in human tissues.^181^ In contrast, DBiT can only map 22 proteins at cellular level resolution.^54^ One drawback for spatial-CITE-seq is the lack of subcellular resolution, a limitation across most spatial multiomics approaches. Other weaknesses for spatial-CITE-seq include competition between ADTs and mRNAs for in-tissue reverse transcription, and poor detection efficiency for low–copy-number transcripts. Protein coverage is also limited to a panel of surface epitopes, excluding intracellular or extracellular matrix proteins, which limits the information provided in regard to protein signaling and function. The major nanotechnologies that support spatial-CITE-seq are ADTs and microfluidic chips with nanometer-wide lanes.

#### 4.5.4 Multi-Omics Single-Scan Assay with Integrated Combinatorial Analysis

MOSAICA is a fluorescence-based spatial multiomics imaging tool for simultaneous codetection of protein and mRNA (Figure 6C).^55^ The MOSAICA procedure uses formalin-fixed tissues or cells, which are incubated with a set of primary oligonucleotide probes that bind to complementary regions (25 to 30 bases long) on mRNAs and contain adapter sequences. After a wash step, a set of secondary probes, each with a pair of fluorophores, binds to the adapters on the primary probes (Figure 6C). Thus, each target has a specific combination of numerous dual-label probes, with emission spectra and temporal lifetime signatures, and these probe maps can be imaged using a fluorescent microscope. Refining these raw data, bioinformatics-based tools direct the reconstruction of images by spectral and fluorescence lifetime signal processing, to allow individual RNAs among a pool of detected targets to be visualized (Figure 6C). These images combine numerous target confocal detections, providing transcript levels and localization within a 3D reconstructed image, which is then laid over the microscopic tissue structure (Figure 6C). MOSAICA has been used for 10-plex mRNA expression in fixed colorectal cancer cells and multiplexed mRNA analysis of clinical melanoma cells within FFPE tissues.^55^ At low cost, MOSAICA achieves high spatial resolution (x-y resolution of 100 nm and z-spacing of 500 nm) in a 3D context (Figure 6C). As an imaging-based tool, MOSAICA suffers from optical crowding, which limits resolution for adjacent targets. But MOSAICA can be integrated with other imaging modalities such as expansion, super-resolution, or multiphoton microscopy to improve subcellular resolution and allow imaging of highly scattering and autofluorescent tissues.^183−185^ In the future, paired fluorescent probes may allow a barcoding strategy based on Förster resonance energy transfer to tune the combinatorial spectrum and lifetime readout.^186^ Nanotechnology supporting MOSAICA includes DNA probes, fluorescent probes, and the wide-field confocal microscope.

## 5 AI and Machine Learning for Spatial Omics in Relation to Nanotechnologies

Dealing with spatial omics data poses significant challenges because of its high dimensionality and complexity and the need for precise spatial and molecular information integration. Recently, more AI-based pipelines and packages have enhanced spatial omics research by enabling the efficient analysis and interpretation of complex biological data at a better resolution.

### 5.1 Workflow of AI-Driven Spatial Omics Profiling

**Figure 7:**
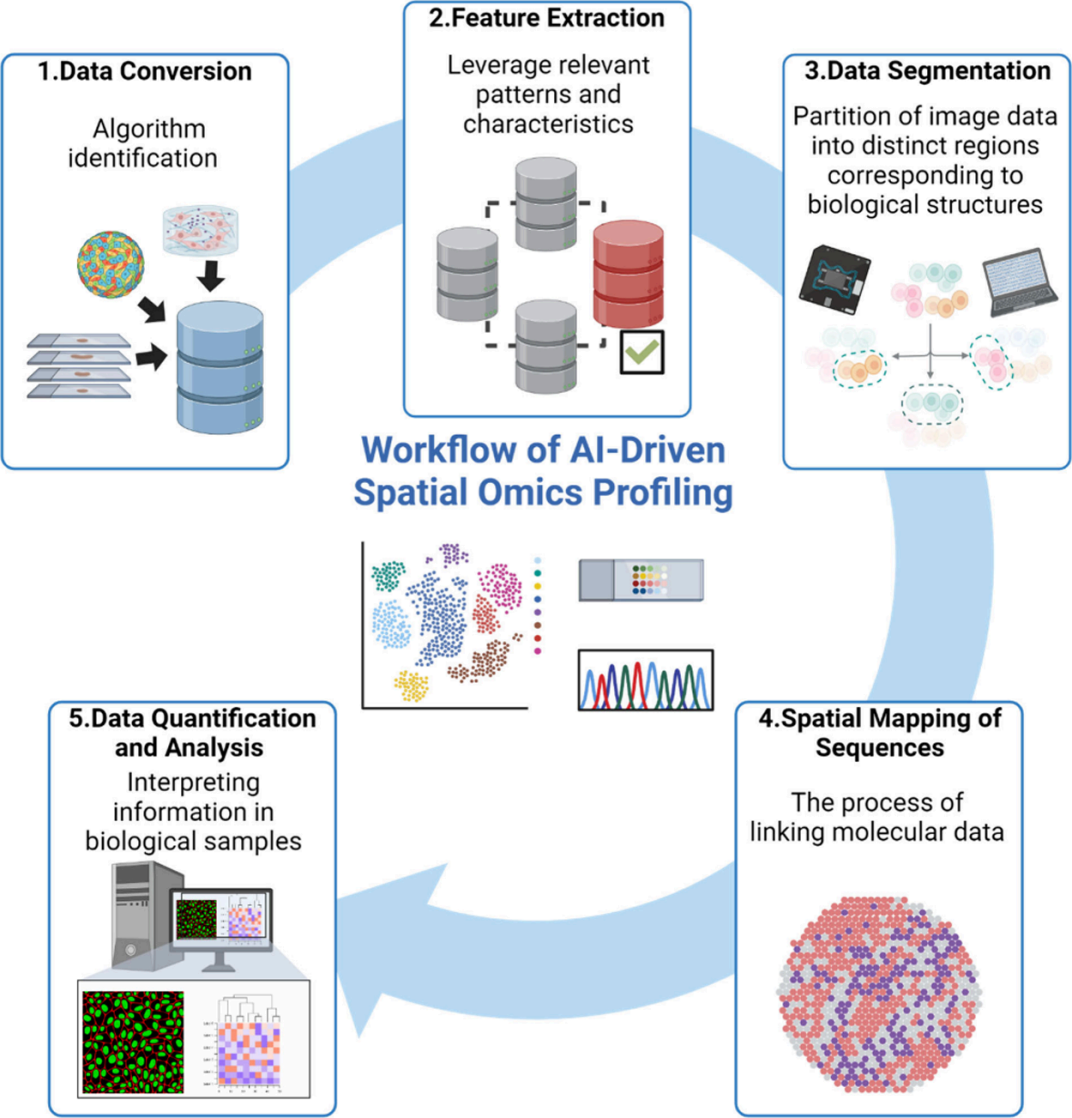
Workflow of AI-Driven Spatial Omics Profiling. The integration of AI into spatial omics follows a structured workflow: data conversion and feature extraction, data segmentation, spatial mapping of sequences, and data quantification and analysis. The figure was created with BioRender.

The integration of AI into spatial omics follows a structured workflow, which includes, in sequential order, data conversion and feature extraction, data segmentation, spatial mapping of sequences, and data quantification and analysis (Figure 7). Data conversion and feature extraction are critical for enabling the algorithm to more effectively identify and leverage relevant patterns and characteristics within the data, such as data indicating gene expression or protein localization, in the context of tissue architecture. Both 2D and 3D serial tissue sections can be computationally aligned and reconstructed for a more detailed and comprehensive view of spatial relationships in tissue architecture.^187^ Data segmentation is a key process that aims to partition image data into distinct regions corresponding to biological structures, such as cells, tissues, or subcellular components. In bioimaging and computer vision, recent AI algorithms have advanced segmentation significantly.^188−190^ Also, accurate segmentation allows researchers to map the spatial distribution of molecular data, such as gene expression or protein localization data, within tissues. Segmentation techniques have shown promise in correlating spatial biomarkers with clinical outcomes. For example, in the context of lung cancer, specific spatial patterns identified through segmentation have been linked to patient responses to treatment, highlighting the importance of segmentation in both research and clinical applications.^191,192^ Spatial mapping of sequences refers to the process of linking molecular data, such as gene sequences or metabolite profiles, to their precise spatial locations within a biological sample. This approach is essential for understanding the spatial organization of complex environments, such as the Tumor Microenvironment (TME), and for understanding how molecular features vary across different regions of tissue. A notable advancement in this area is the development of the Single-Cell Spatially Resolved Metabolic (scSpaMet) pipeline, which identifies a wide range of metabolites alongside multiplex protein analysis. This detailed mapping is crucial for studying the TME, as it can reveal how different cells and molecules interact, influence tumor progression, or therapy response.^193^ Data quantification and analysis play critical roles in accurately interpreting the complex molecular and cellular information inherent in biological samples. For handling disaggregated data, various computational tools have been developed, including many popular packages such as Seurat, ScateR, Scanpy, and Monocle, allowing researchers to explore omics data down to the single-cell level, which facilitates insights into cellular composition, gene expression, and spatial distribution.^194−196^ On the other hand, a geometric deep learning framework, PINNACLE, has been designed to generate context-aware protein representations that leverage how proteins interact within their cellular environment.^197^ AI techniques have rapidly advanced the spatial omics field by enabling more efficient analysis and breakthroughs in understanding tissue architecture and disease mechanisms. As AI pipelines continue to evolve, they are beginning to extend into the nanotechnology field, where similar techniques can be applied to nanoscale biological structures, further pushing the boundaries of molecular and cellular research.

### 5.2 AI for Nanometer-Scale Data Processing

The advent of nanotechnology has allowed researchers to elucidate biological structures and functions at a better resolution, often at the nanometer scale. For example, super-resolution microscopy techniques, such as Stimulated Emission Depletion (STED) microscopy,^198^ enable the visualization of biomolecules at a resolution beyond the diffraction limit. However, the data generated from such techniques are vast and complex, necessitating sophisticated AI tools for their analysis.

Deep learning models, particularly Convolutional Neural Networks (CNNs), have proven highly effective in handling high-resolution spatial omics data. CNNs are widely used for image analysis tasks such as feature extraction, segmentation and pattern recognition in large data sets.^190^ In this context, CNNs automate the detection of intricate spatial features within biological tissues, providing insights into tissue architecture that are otherwise difficult to uncover. The application of U-Net, a CNN architecture specifically designed for biomedical image segmentation, has further enhanced our ability to extract meaningful data from nanometer-scale images.^190^ CNNs play a crucial role in the structured workflow, especially in the feature extraction and segmentation stages, where they capture spatial features hierarchically from 2D images.^199^ Moreover, deep learning models enable the reconstruction of 3D structures from serial tissue sections, advancing our understanding of spatial relationships within tissues.^187^ AI also supports the transition from 2D to 3D analysis, allowing for the reconstruction of spatial relationships across tissue volumes.^187^ For example, K-means clustering is commonly used for sequence-to-location mapping in 3D tissue profiling, helping to delineate cell populations within tissue architectures.^193^ Advanced AI models such as CODA have been developed to visualize 3D tissue architecture in large tissue samples. These models enable the discovery of cell types and their spatial organization in tissues such as the skin, lungs, and liver.^200^

### 5.3 Machine Learning for Multi-Omics Data Integration

Integrating data across multiple omics layers—spatial transcriptomics, proteomics, and metabolomics—is key to understanding the complex interactions governing tissue function. Machine learning techniques, such as random forests and Support Vector Machines (SVMs), facilitate this integration by identifying correlations across data sets.^201^ These methods have been employed in spatial omics to merge multiomics data, thereby revealing the intricate dynamics of cellular environments and tissue-specific processes.^202^

Recent advancements in graph-based machine learning approaches, such as Graph Convolutional Networks (GCNs), have further improved our ability to integrate spatial information with multiomics data. For instance, SpaGCN combines spatial transcriptomics data with histological information to identify spatial domains and variable genes in tissues.^203^ Additionally, unsupervised learning approaches like Graph-Based Convolutional Networks (DSTG) have been developed to deconvolute spatial transcriptomics data, helping to uncover underlying biological relationships.^204^

### 5.4 Nanotechnology and AI for Spatial Biomarker Discovery

The integration of nanotechnology with AI has revolutionized spatial biomarker discovery, particularly in the context of disease research. By leveraging nanostructure-labeled targets, such as DNA nanostructures, and coupling them with AI-based analytical tools, researchers can identify spatial patterns that are otherwise indiscernible. For example, nanotechnology-enhanced methods combined with AI-driven models have facilitated the detection of biomarkers in cancer tissues, leading to a better understanding of tumor heterogeneity and progression.^205^

AI also plays a pivotal role in interpreting spatial omics data obtained from nanodevices, such as nanoparticle-based sensors and nanoscale imaging probes. These devices capture high-resolution molecular information that, when processed by AI models, reveals spatially resolved biomarker patterns linked to clinical outcomes.^191^ The application of deep learning techniques, such as geometric deep learning used in PINNACLE, generates context-aware protein representations, offering avenues for precision medicine.^197^

## 6 Challenges and Opportunities Ahead

Methods and techniques from the nanotechnology field have shaped spatial omics to allow insight into biological systems on the nanometer scale, providing analyses unfeasible before advances in nanotechnological methods. The intrinsic properties of nanomaterials, coupled with methodologies using nanodevices and nanobiotechnological tools, enable precise cellular and subcellular labeling and sequencing. The goal of spatial omics is, ultimately, to provide real-time, in situ multiomics measurement of biomolecules at a resolution necessitated by the specific application, which for some applications, may be nanometer resolution. But as evident through this review, inherent hurdles within spatial omics must be resolved to accomplish this goal.

Relying on PCR-based nucleotide amplification for sequencing in transcriptomics must be overcome to prevent amplification bias and spatial context loss while improving dynamic range and throughput. While DNA nanoballs have helped to an extent, they do not improve dynamic range or eliminate sequence bias, both of which are inherent in various methods of nucleic acid amplification.^206^ To date, only de-novo sequencing has eliminated amplification bias due to its specific chemistry and is both highly sensitive for single molecule nucleic acid sequencing and independent of PCR.^155^ Consequently, other nanotechnologies, most notably nanopore sequencing,^149,150^ have flourished in this area. Initially developed for the stochastic sensing of ions and small molecules, nanopores act as single-molecule biosensors, facilitating ultrasensitive DNA sequencing in comparison to other label-free biomolecular sensing techniques.^207−209^ The commercially available nanopore sequencer, MinION, is a nanodevice that employs a protein pore residing in an electrically resistant polymer membrane, exemplifying lab-on-a-chip potential.^210−212^ And rapid advances in nanopore technologies for sequencing long molecules of DNA and RNA have helped investigate genomes, transcriptomes, epigenomes, and epitranscriptomes.^213,214^ Future nanopore developments may enable miniaturized RNA sequencing via geometric sensitive current disruptions, applied in direct contact with tissue, improving detection sensitivity and accuracy at subcellular resolution.^5,78^ Synergizing these nanopores with imaging tools could help to further advance spatial transcriptomics and spatial epigenomics. Additionally, nanopores may prove useful for spatial proteomics, for de novo protein and peptide sequencing.^215^

While current research trajectories aim to combine spatial proteomics with nanotechnology, spatial proteomics’ reliance on antibody binding for protein detection defines an inherent limit for protein or peptide coverage. The most widely used instrument in proteomics, mass spectrometry, cannot analyze peptide sequences directly without relying on antibodies. One promising avenue is using nanomaterial matrices to enhance MALDI signals, which can dramatically improve MALDI resolution.^216^ Improving the sensitivity of MALDI for spatial proteomics would allow for detection that is free from artifacts due to antibody-based selection or detection and would thereby increase the breadth of protein coverage in a step toward whole proteome analysis. Enhancing metabolomic and lipidomic coverage is also possible by similarly applying nanomaterial matrices to enhance SIMS signals.^217^

In terms of resolution for protein identification, liquid chromatography-mass spectrometry (LC-MS) is superior to all other techniques, but an inherent limitation prevents the application of LC-MS to spatial proteomics: low sample throughput resulting from lengthy processing times. Analyzing the spatial transcriptome—2500 assay points within a 6.5 mm by 6.5 mm section—can be completed in a reasonable time frame. But comparable proteomic analysis, with similar coverage by LC-MS and assuming 30 min per protein target, would take two months for a single histological section, not including sample pretreatment. Microfluidic platforms with embedded nanoscale features significantly increase the speed of sample preprocessing while automating batch sample processing and reducing scale for enhanced resolution. Microfluidics technology is already used in single-cell proteomics,^218^ and it is only a matter of time before this technology is applied to spatial proteomic applications. Microfluidic sample processing would still have the problem of lengthy LC-MS assay times, but barcoding technologies could multiplex protein labeling and facilitate analysis of up to 16 samples at a time. Among nanobiotechnologies, barcoding has had the greatest impact, notably for its ability to analyze >20,000 barcodes simultaneously. EEL FISH, for example, exploits combinatorial barcodes that can label thousands of targets after only 16 rounds of detection, representing a rudimentary example of nanocomputing. Proteomics methods that similarly analyze more than 20,000 barcoding tags simultaneously, as in transcriptomics, would solve the detection throughput bottleneck for this biomolecular domain.

Once nanotechnology and nanomaterials have been fully utilized to increase the resolution and data throughput of spatial-omics studies, we can anticipate an exponential increase in the amount of generated data. Thus, analyzing these vast data sets while combining the results of multiomics studies in coherent ways represents a corollary challenge for spatial multiomics. Most current spatial omics focus on the 2D level. The ability to integrate data from multiple planes and time periods to create 3D images represents a wider challenge but would also be a major breakthrough for the future of spatial omics. SIMS-mediated approaches toward spatially resolved 3D metabolomics are already in development,^193,219^ and it will be exciting to see how lessons learned from these technologies can be adapted to other spatial omics domains.

Another domain that needs to be addressed by spatial omics tools is the secretome, circulating molecules including proteins, lipids, and vesicles secreted by cells. Spatial secretomics has barely moved forward due to a lack of tools that can accurately locate and discriminate between internal and external cellular components. Nanoscale liposomes have been used to characterize the nucleic acid composition inside extracellular vesicles,^220^ and it would be exciting to study how these liposomes could be used to analyze extracellular vesicle components on sections, including the spatial distribution of these components. Extracellular vesicles can be used as diagnostic markers to reveal information underlying disease development, such as spatial interactions between pathogens and immune cells in infectious diseases, signals predicting tumor cell metastasis in cancer, and early changes in lesions to predict severity and progression in neurological diseases.

As spatial omics methods develop, the resulting data will increase in complexity, and more AI and machine learning studies will be required. Integrating nanotechnology-enhanced methods, combined with AI-driven models, will benefit the spatial omics results to achieve nanoresolution. AI and machine learning are necessary for fully utilizing nanotechnology and nanomaterials in spatial-omics studies.

In summary, the development of research methods for spatial multiomics is flourishing with the support of nanotechnology, but bottlenecks prevent in situ, real-time multiomics from being achieved. Increasing the sensitivity, breadth of coverage, and resolution of spatial omics tools by leveraging emerging nanotechnologies will certainly help to improve spatial omics. These powerful tools are helping biomedical science to further elucidate physiological structure and function, and they provide superior diagnostic and therapeutic tools for disease research.

## Abbreviations

2D: two-dimensional
3D: three-dimensional
ADTs: Antibody-Derived DNA Tags
BaristaSeq: Barcode in situ Targeted Sequencing
BARseq: Barcoded Anatomy Resolved by Sequencing
cDNA: complementary DNA
CID: Coordinate Identity
CITE: Co-Indexing of Transcriptomes and Epitopes
CNNs: Convolutional Neural Networks
CODEX: CO-Detection by Indexing
CT: Corticothalamic
CUT&Tag: Cleavage Under Targets and Tagmentation
CyToF: Cytometry by Time-of-Flight
DBiT-seq: Deterministic Barcoding in Tissue for Spatial Omics Sequencing
DESI: Desorption Electrospray Ionization
dsDNA: double-stranded DNA
DSP: Digital Spatial Profiler
DSTG: Graph-Based Convolutional Networks
EEL FISH: Enhanced Electric FISH
FFPE: Formalin Fixed Paraffin Embedded
FISSEQ: Fluorescent in situ RNA Sequencing
FRET: Förster Resonance Energy Transfer
GCNs: Graph Convolutional Networks
HDMI-array: High-Definition Map Coordinate Identifier-Encoded Rna-Capturing Array
IT: Intratelencephalic
ITO: Indium Tin Oxide
LCM: Laser Capture Microdissection
LC-MS: Liquid Chromatography Mass Spectrometry
LDI MS: Laser Desorption/Ionization MS
lncRNA: long noncoding RNA
MALDI: Matrix-Assisted Laser Desorption/Ionization
MAPseq: Multiplexed Analysis of Projections by Sequencing
MHC: Major Histocompatibility Complex
MIBI: Multiplexed Ion Beam Imaging
MOSAICA: Multi-Omics Single-Scan Assay with Integrated Combinatorial Analysis
MOSTA: Mouse Organogenesis Spatiotemporal Transcriptomic Atlas
mRNA: mRNA
miRNA: microRNA
MS: Mass Spectrometry
MSI: Mass Spectrometry Imaging
nano-DESI: Nanospray Desorption Electrospray Ionization
PCR: Polymerase Chain Reaction
PDMS: Polydimethylsiloxane
PT-like: Pyramidal Tract-like
RCA: Rolling-Circle Amplification
scDVP: Single-Cell Deep Visual Proteomics
scRNA-seq: Single-Cell RNA Sequencing
scStereo-seq: Single-Cell Stereoseq
scSpaMet: Single-Cell Spatially Resolved Metabolic
SEDAL: Sequencing with Error-Reduction by Dynamic Annealing and Ligation
SIMS: Secondary Ion Mass Spectrometer
SIMS Imaging: Secondary Ion Mass Spectrometry Imaging
siRNA: small interfering RNA
smFISH: Single-Molecule RNA Fluorescence in situ Hybridization
SNAIL: Specific Amplification of Nucleic Acids via Intramolecular Ligation
snoRNA: small nucleolar RNAs
snRNA: small nuclear RNAs
SNV: Single-Nucleotide Polymorphism
spatial-ATAC-seq: Spatially Resolved Assay for Transposase Accessible Chromatin Profiling of Tissue Sections using Next-Generation Sequencing
spatial-CITE-seq: Spatial Co-Indexing of Transcriptomes and Epitopes for Multi-Omics Mapping by Highly Parallel Sequencing
STARmap: Spatially Resolved Transcript Amplicon Readout Mapping
Stereoseq: Spatial Enhanced Resolution Omics-Sequencing
STED: Stimulated Emission Depletion
SVMs: Support Vector Machines
TME: Tumor Microenvironment
ToF: Time of Flight
UMI: Unique Molecular Identifier

## Vocabulary

Spatial omics: is a method capable of describing biomolecule information layered onto histological landscapes and refines structure/function definition to almost 1 nm.
Rolling-circle amplification (RCA): is an isothermal nucleic acid amplification method widely applied for in vivo imaging of various targets, including mRNA, dsDNA, miRNA, and proteins.
DNA nanoball: nanometer-sized DNA balls comprise RCA-produced copies of a specific sequence used to amplify specific detection signals for imaging.
Super-resolution microscopy: coupled with fluorescent-labeled probes allows the acquisition of high-resolution images of target sequences in the nanometer range.
DNA barcode: comprises a specific sequence tag covalently attached to biomolecular targets and designed to be excluded from the sequence domain of the species queried; its identity allows measurements to be linked to specific spatial or temporal locations.
Mass Spectrometry Imaging (MSI): is a primary nanotechnology tool that uses mass/charge information to identify proteins and metabolites and allows for visualization of spatial distributions in various samples.
